# Dual‐Modular Hydrogel Microparticles with Precision‐Modulation of Inflammatory Microenvironment Dictate Full‐Thickness Cartilage Regeneration for Osteoarthritis Repair

**DOI:** 10.1002/advs.202504965

**Published:** 2025-06-29

**Authors:** Xinye Chen, Yuanman Yu, Zirui He, Lina Pan, Jing Wang, Changsheng Liu

**Affiliations:** ^1^ The State Key Laboratory of Bioreactor Engineering East China University of Science and Technology Shanghai 200237 China; ^2^ Key Laboratory for Ultrafine Materials of Ministry of Education East China University of Science and Technology Shanghai 200237 China; ^3^ Frontiers Science Center for Materiobiology and Dynamic Chemistry East China University of Science and Technology Shanghai 200237 P.R. China

**Keywords:** cartilage regeneration, hydrogel microparticles, inflammatory modulation, osteoarthritis

## Abstract

Osteoarthritis (OA) is a typical degenerative disease characterized primarily by the degeneration of cartilage. However, current treatments for cartilage degeneration often lead to the formation of hypertrophic cartilage and fibrocartilage, making it challenging to achieve full‐thickness cartilage. Here, dual‐modular hydrogel microparticles (dmHMPs) are developed, which enable precise spatio‐temporal modulation and dynamic equilibrium between immune responses and cartilage regeneration. dmHMPs can modulate the inflammatory microenvironment of the joint by promoting the polarization of synovial macrophages toward an anti‐inflammatory phenotype. Consequently, as inflammation is mitigated, synovium‐derived mesenchymal stem cells recruited by dmHMPs can efficiently undergo chondrogenic differentiation to repair damaged cartilage in situ. In vivo experiments demonstrate that dmHMPs significantly suppress the expression of Indian hedgehog (Ihh) and collagen type X, thereby reducing the formation of hypertrophic cartilage and preserving the structural integrity and biological functions of articular cartilage. This therapeutic modulates the dynamic balance between inflammation and repair, providing a promising approach for full‐thickness cartilage regeneration.

## Graphical abstract

Cartilage injury is a prominent pathological hallmark of osteoarthritis. In this study, we developed a two‐modular hydrogel microparticle system wherein HMP‐A effectively targets the damaged area, while HMP‐B forms an efficient connection with HMP‐A on the cartilage surface. This dual‐modular system can significantly promote full‐thickness cartilage regeneration by modulating inflammation and recruiting SMSCs.

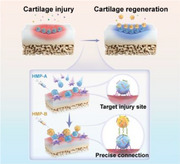

## 1 Introduction

Osteoarthritis (OA) is a prevalent degenerative disease caused by various factors, including aging,^[^ ^1^ ^]^ obesity,^[^ ^2^ ^]^ and trauma.^[^ ^3^ ^]^ This disease leads to a substantial reduction in patient mobility and affects billions of individuals globally, imposing a considerable burden on both patients and society due to its widespread prevalence and associated economic costs.^[^ ^4^ ^]^ As cartilage injuries are generally associated with the progressive onset of OA, robustly effective approaches for cartilage regeneration are necessary.^[^ ^5^, ^6^, ^7^ ^]^ However, the avascular and aneural characteristics of articular cartilage limit its nutrient supply, significantly constraining its regenerative capacity.^[^ ^8^, ^9^ ^]^ Despite extensive research and development efforts, progress in cartilage regeneration has been relatively slow, partly due to the neglect of the complex pathological environment during OA progression, including mechanical changes,^[^ ^10^ ^]^ chronic inflammation,^[^ ^11^, ^12^ ^]^ and metabolic alterations.^[^ ^13^, ^14^, ^15^ ^]^ Current treatment strategies predominantly result in fibrocartilage or hypertrophic cartilage repair, both of which exhibit markedly inferior biomechanical properties compared to normal hyaline cartilage.^[^ ^16^, ^17^ ^]^ To date, no surgical, material, cellular, or pharmacological interventions can reliably and durably restore the structural integrity and functional characteristics of hyaline cartilage.^[^ ^18^ ^]^ Therefore, developing effective strategies for regenerating and restoring the complete biological function of hyaline cartilage is crucial for delaying the progression of OA.

Various factors contribute to the failure of cartilage regeneration, with uncontrolled inflammatory responses considered a primary contributor throughout the OA repair process.^[^ ^19^ ^]^ Synovitis is a key driver of inflammatory progression in OA, wherein synovial macrophages serve as critical mediators.^[^ ^20^ ^]^ Upon the onset of OA, the synovium becomes inflamed, leading to the activation of pro‐inflammatory macrophages. These macrophages secrete a plethora of inflammatory cytokines, including IL‐1β and TNF‐α, as well as chemokines such as CCL2 and CCL4, thereby accelerating OA pathogenesis.^[^ ^12^ ^]^ Nevertheless, the invasion of synovium plays a dual role in the repair processes associated with OA, as it also facilitates the recruitment of synovium‐derived mesenchymal stem cells (SMSCs). SMSCs are well‐documented for their substantial regenerative potential, attributed to their capacity to differentiate into chondrocytes and secrete extracellular matrix components essential for cartilage repair.^[^ ^21^, ^22^, ^23^ ^]^ However, the availability of SMSCs is limited, and their chondrogenic capacity often diminishes while exhibiting a tendency toward fibrogenesis due to peripheral pro‐inflammatory synovial macrophage influence.^[^ ^24^ ^]^ To address the challenges associated with utilizing endogenous SMSCs for advancing OA cartilage repair, we propose that the treatment strategy should possess the following characteristics: a) precise manipulation of the inflammatory microenvironment to foster repair; b) efficient recruitment of SMSCs to the defects; c) meticulous orchestration between immune regulation and chondrogenic differentiation. Moreover, the immune response and regenerative repair constitute a sequential cascade process, wherein immune regulation plays a guiding role in the subsequent repair mechanisms. Therefore, it is imperative to devise a dual‐modular system that can adapt to this dynamic progression.

**Figure 1:**
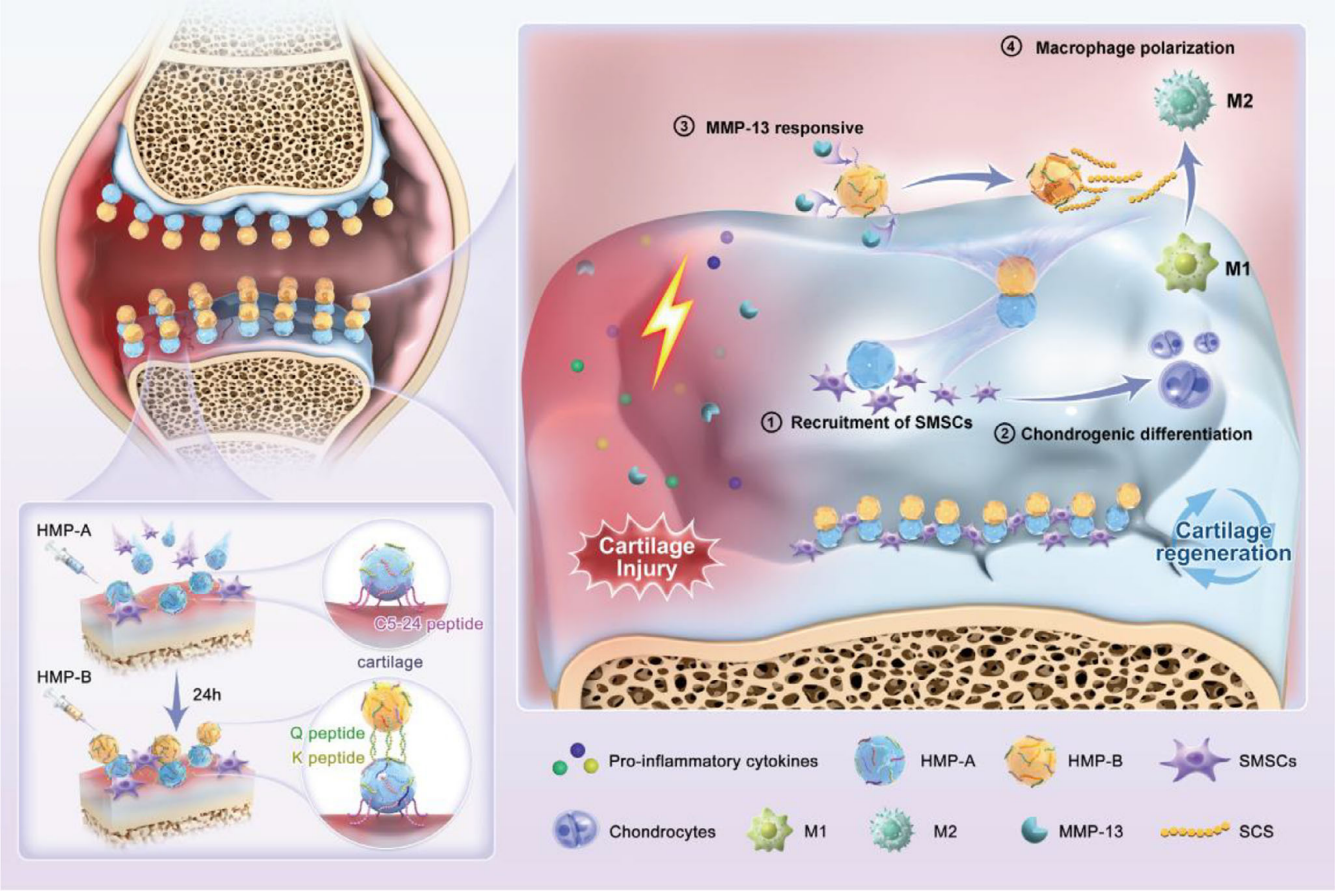
Schematic illustration of the dual‐modular hydrogel microsphere system (dmHMPs) for OA treatment. HMP‐A and HMP‐B were sequentially injected into the joint cavity at a 24‐hour interval. This approach aimed to minimize the steric hindrance caused by the premature interaction between the two HMPs, thereby preserving the targeting efficiency of HMP‐A for damaged cartilage and establishing an orderly two‐module structure. HMP‐A targeted to the injured cartilage area via the C5‐24 peptide, and subsequently, the two HMPs self‐assemble through K and Q peptide interactions, forming a dmHMP structure. HMP‐A and HMP‐B exhibit distinct biological functions in OA treatment. HMP‐A targets the damaged cartilage area and recruits SMSCs. HMP‐B, responds to MMP‐13, rapidly releasing SCS to regulate the inflammatory environment and promote the polarization of synovial macrophages from pro‐inflammatory M1 to anti‐inflammatory M2 phenotypes. Together, dmHMPs synergistically regulate the pathological microenvironment to enhance cartilage regeneration and alleviate OA symptoms.

In this study, we developed an advanced dual‐modular system leveraging hyaluronic acid (HA) hydrogel microparticles (HMPs) to enhance cartilage regeneration in OA treatment, capitalizing on their hydration and lubrication properties to preliminary alleviate cartilage wear and pain.^[^ ^25^, ^26^ ^]^ The system comprises two microparticles, namely HMP‐A and HMP‐B. HMP‐A specifically targets damaged cartilage through the collagen‐targeting peptide C5‐24, facilitating the recruitment of SMSCs and inducing their differentiation into chondrocytes via the SKPPGTSS peptide (SKP peptide), thereby initiating cartilage repair (**Figure** **1**). Subsequently, HMP‐B is anchored to HMP‐A through K peptide and Q peptide interaction, mimicking fibrinogen–thrombin binding. Designed to respond rapidly to the MMP‐13 enzyme present in the inflammatory environment near joint injuries, HMP‐B releases sulfated chitosan (SCS), a heparin‐like polysaccharide that effectively shifts macrophage polarization from pro‐inflammatory M1 to anti‐inflammatory M2 state, enhancing the secretion of reparative factors.^[^ ^27^, ^28^ ^]^ The effective immunoregulation inhibits SMSCs differentiation into fibrosis and improves the OA repair quality. Through the intelligent design and in situ construction of this dual‐modular HMP system (dmHMPs), biomaterial‐mediated OA repair can be accomplished with enhanced precision at sites of cartilage injury, effectively addressing localized damage. dmhMPs significantly downregulated the expression of the cartilage hypertrophy marker Indian hedgehog protein (Ihh) and the fibrocartilage marker collagen type I (Col I), thereby facilitating the regeneration of full‐thickness articular cartilage. This ultimately resulted in effective treatment of degenerative OA, even in an aged microenvironment.

## 2 Results

### 2.1 Preparation and Characterization of HMPs

To facilitate further modification and crosslinking of hydrogel microparticles, we initially subjected HA to chemical modification using methacrylic anhydride (MA), resulting in the synthesis of hyaluronic acid methacrylate (HAMA). By introducing a double bond through grafting, HA becomes capable of undergoing Michael addition reactions with subsequently added peptides containing sulfhydryl groups. The successful grafting of MA was confirmed by proton nuclear magnetic resonance (^1^H NMR) spectra (Figure S1A, Supporting Information). To determine the appropriate concentration of HAMA solution for hydrogel microparticle preparation, we evaluated the viscosities of HAMA solutions at three distinct concentrations: 2.5, 5, and 10 wt.% (Figure S1B, Supporting Information). With the increase of concentration, the viscosity of the solution also increases significantly. However, if the viscosity of the dispersed phase is too high, it will cause high pressure in the microchannels, which is not conducive to the generation of microparticles. Therefore, we chose a 5wt% HAMA solution with moderate viscosity.

**Figure 2:**
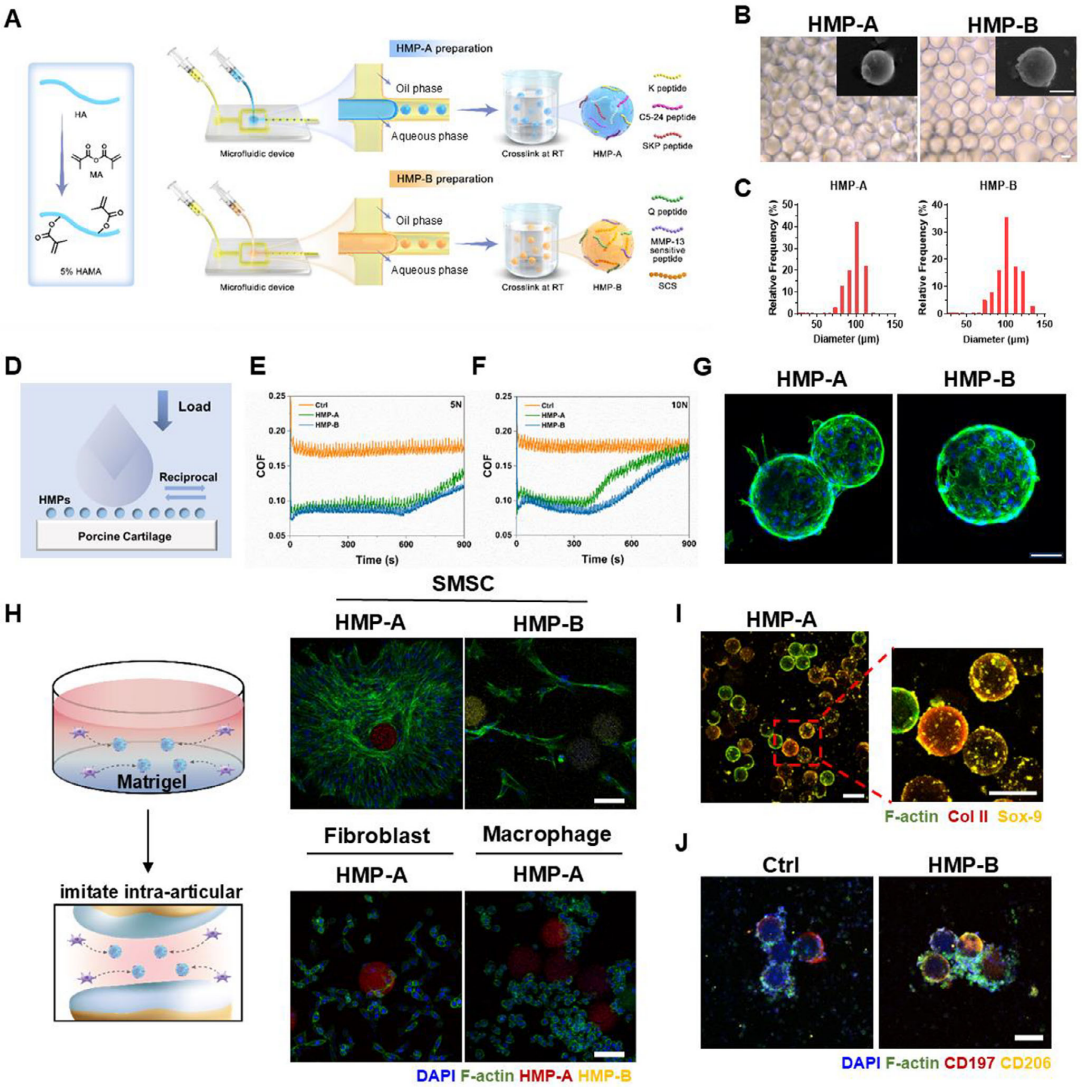
Preparation and characterizations of HMP‐A and HMP‐B. A) Schematic representation of the preparation process for HMPs. B) Representative bright‐field images and scanning electron microscopy (SEM) images of HMP‐A and HMP‐B. Scale bar, 50 µm. C) Size distribution of HMP‐A and HMP‐B. D) Schematic illustration of tribological test of HMPs. E,F) COF–time curve for Ctrl, HMP‐A, and HMP‐B under 5 and 10 N, respectively. G) Cell adhesion of chondrocytes co‐cultured with HMP‐A and HMP‐B for 7 days. F‐actin was stained with rhodamine‐phalloidin, and nuclei were stained with DAPI. Scale bar, 50 µm. H) Schematic and fluorescence images of selective recruitment of SMSC in vitro. HMP‐A was labeled with Rhodamine. HMP‐B was labeled with AF‐488. Scale bar, 100 µm. I) Representative images of Col II and Sox‐9 immunostainings of SMSCs cultured on HMP‐A for 7 days. Scale bars, 100 µm. J) Representative images of CD197 and CD206 immunostainings of LPS‐induced macrophages cultured on HMP‐B for 3 days. Scale bars, 100 µm.

To address the complex pathological microenvironment encountered during OA repair, we developed two types of HMPs, designed as HMP‐A and HMP‐B. These HMPs, differing in composition, were fabricated using a microfluidic water‐in‐oil emulsion technique (**Figure** **2**A). Crosslinking of the hydrogel droplets was performed at room temperature, resulting in the formation of hydrogel microparticles. The Michael addition reaction was utilized, involving the double bonds of the sulfhydryl group and HAMA at the cysteine termini of the SKP peptide and MMP‐13 sensitive peptide, respectively. The microfluidic method offers several advantages over other preparation techniques, including uniform composition and size distribution, thereby greatly facilitating subsequent in vivo and in vitro experimental investigations. The morphology of the microparticles was characterized using an optical microscope and scanning electron microscope (SEM), revealing good dispersion, uniform structure, and consistent size in aqueous solution. Additionally, SEM images demonstrated that the surface of the lyophilized microparticles was smooth, thereby augmenting their lubrication efficacy during later in vivo stages (Figure 2B). Elemental analysis and mapping were performed using energy‐dispersive spectroscopy (EDS) to determine the distribution of elements within the HMPs. The results indicated that carbon (C), nitrogen (N), oxygen (O), and sulfur (S) elements were evenly distributed, confirming the uniform composition of the HMPs (Figure S1C, Supporting Information). Previous studies have demonstrated that HMPs with a particle size of ≈100 µm are advantageous for lubrication and the treatment of OA.^[^ ^29^, ^30^, ^31^ ^]^ Consequently, in this study, HMP‐A and HMP‐B were prepared with a particle size of ≈100 µm to align with these findings. Both HMP‐A and HMP‐B exhibited particle sizes of ≈100 ± 12.3 µm (Figure 2C), with a narrow size distribution. Notably, HMP‐B had a slightly larger average size than HMP‐A (Figure 2C), potentially due to the increased density resulting from the presence of SCS.

Given the critical role of joint lubrication in the treatment of OA, we initially examined the lubricating capacity of HMPs. Both HMP‐A and HMP‐B underwent a 900‐second friction process assessment using a universal materials tester (UMT‐3). In this procedure, porcine cartilage was employed to simulate the lubrication properties of the microparticles on the cartilage surface. A reciprocating friction test was applied to the material under forces of 5 and 10 N, respectively (Figure 2D). The tribological results revealed that both HMP‐A and HMP‐B exhibited effective lubrication under a pressure of 5 N (Figure 2E). However, under a pressure of 10 N, HMP‐B demonstrated superior lubrication performance compared to HMP‐A (Figure 2F). These findings underscore the potential of HMP‐B to provide effective lubrication under higher mechanical loads, which is crucial for its application in OA treatment.

Next, to assess the biocompatibility of HMP‐A and HMP‐B, we employed a co‐culture system to evaluate the chondrocytes proliferation and adhesion. Live‐Dead analysis indicated good cell viability across all time points (days 1, 4, and 7), with no significant differences between the groups (Figure S2, Supporting Information). The co‐culture of chondrocytes and the two types of HMPs demonstrated that the HMPs facilitated chondrocyte adhesion and growth, with the cells on the surface of the microparticles displaying a favorable spreading morphology (Figure 2G). Thus, HMPs enhanced the proliferation, cell viability, and adhesion of SMSCs, reflecting a satisfactory ability to maintain stem cell numbers and viability.

Previous studies have demonstrated the remarkable recruitment abilities of the SKP peptide for SMSCs,^[^ ^22^ ^]^ a finding that was further confirmed by our transwell assay (Figure S3, Supporting Information). The cell migration results in the transwell assay indicated that SKP peptide and HMP‐A loaded with SKP peptide could effectively recruit SMSCs within 24 h, and the number of recruited cells was four times that of the control group. Consequently, we employed HMP‐A conjugated with the SKP peptide to effectively recruit SMSCs to cartilage defects. Immunostainings showed that HMP‐A had a significant recruitment effect on SMSCs compared with HMP‐B (Figure 2H). Interestingly, in addition to efficiently promoting the recruitment of SMSCs, HMP‐A displayed selective cell recruitment. When co‐cultured with matrix gel containing HMP‐A, there was a noticeable migration trend of SMSCs toward it as opposed to macrophages and fibroblasts, indicating the excellent selective recruitment ability of HMP‐A for SMSCs. Meanwhile, we quantitatively compared the fluorescence intensities of macrophages and SMSCs in the surrounding area of HMP‐A (Figure S4, Supporting Information). The results showed that the fluorescence intensity of SMSCs was 5 to 10 times higher than that of macrophages. It suggested that HMP‐A exhibits a significant recruitment effect on SMSCs, whereas the macrophages around HMP‐A are distributed randomly and do not demonstrate a notable recruitment effect.

Another crucial aspect to consider is whether the recruited SMSCs can undergo smooth differentiate into chondrocytes. As previous studies have reported that employing HMPs as a 3D scaffold facilitated chondrogenic differentiation of stem cells and enhanced the formation of cartilage matrix,^[^ ^32^, ^33^ ^]^ our initial investigation focused on evaluating the impact of SMSC culture in both 2D and 3D systems on chondrogenic differentiation. Immunofluorescence staining revealed that culturing SMSCs on HMPs significantly enhanced the expression of chondroid‐related markers Col II and Sox‐9 compared to conventional 2D substrates (Figure 2I; Figure S5A–C, Supporting Information). Additionally, Alcian blue staining and Safranine O staining revealed a significant increase in glycosaminoglycan (GAG) expression on the surface of hydrogel microparticles (Figure S5D, Supporting Information), thereby providing further confirmation of the enhanced promotion of chondrocyte differentiation on HMPs. To further investigate the effect of HMPs on chondrogenic differentiation of SMSCs, we assessed this phenomenon by subjecting cartilage pellets to a 14‐day culture supplemented with chondroblast differentiation medium. All pellets were subjected to staining with toluidine blue, Alcin blue, and Col II immunofluorescence. Notably, the pellet treated with HMP‐A and dmHMPs exhibited enhanced GAG synthesis and Col II expression (Figure S6A–C, Supporting Information). Thus, HMPs can significantly enhance the chondrogenic differentiation of SMSCs and promote the generation of glycosaminoglycans in the extracellular matrix of chondrocytes.

To further elucidate the mechanism by which hydrogel microspheres regulate the differentiation of SMSCs into chondrocytes, we examined the influence of HMPs on integrin‐mediated signaling pathways, with a focus on integrin α5β1 due to its established role in modulating chondrogenesis and downstream cartilage matrix synthesis.^[^ ^34^ ^]^ Immunostainings revealed that HMP‐A significantly upregulated the expression of surface integrin α5β1 on SMSCs (Figure S7A–D, Supporting Information). Furthermore, both immunofluorescence and Western blot analyses confirmed that HMPs sustained the activation of downstream signaling pathways, including Focal Adhesion Kinase (FAK) and protein kinase B (AKT) (Figure S7E–G, Supporting Information). To validate the critical role of integrin signaling in the HMP‐mediated regulation of chondrogenic differentiation, we utilized Cilengitide to inhibit integrin α5β1 signaling in SMSCs. Notably, this inhibition effectively abolished the activating effects of HMPs on FAK and AKT phosphorylation, leading to a marked reduction in the expression of the downstream transcription factor Sox‐9 (Figure S7H, Supporting Information). This decrease in Sox‐9 levels was closely associated with reduced synthesis of cartilage matrix‐associated proteins. Collectively, these findings demonstrate that the regulatory effects of HMPs on the differentiation of SMSCs into chondrocytes are predominantly mediated by integrin α5β1 signaling.

During the initial design phase, HMP‐B was anticipated to exhibit a targeted response toward the heightened expression characteristics of MMP‐13 on defective cartilage surfaces, thereby facilitating the controlled release of immunoregulatory adjuvant SCS and enabling precise modulation of the immune microenvironment surrounding OA cartilage. Our previous studies have demonstrated that SCS effectively regulates the polarization of macrophages from the pro‐inflammatory M1 phenotype to the anti‐inflammatory M2 phenotype, leading to a reduction in pro‐inflammatory factor expression within the inflammatory microenvironment.^[^ ^27^, ^35^ ^]^ To assess whether the HMP‐B‐released SCS retains the immunomodulatory ability, LPS‐stimulated macrophages were co‐cultured with HMP‐B, and subsequent evaluation was performed on the expression of M1 and M2 markers. Immunostainings revealed a significant increase in the number of CD206‐positive macrophages in the HMP‐B compared to the Ctrl group (Figure 2J), indicating that encapsulation of SCS within HMP‐B can effectively regulate the phenotypic transformation of macrophages from M1 to M2 under inflammatory conditions. Western Blots analysis indicated that HMP‐B promoted the polarization of macrophages toward M2 by activating the phosphorylation of STAT6 (Figure S8, Supporting Information).

### 2.2 Enhanced Lesion Localization with an In Situ Assembly into dmHMPs

**Figure 3:**
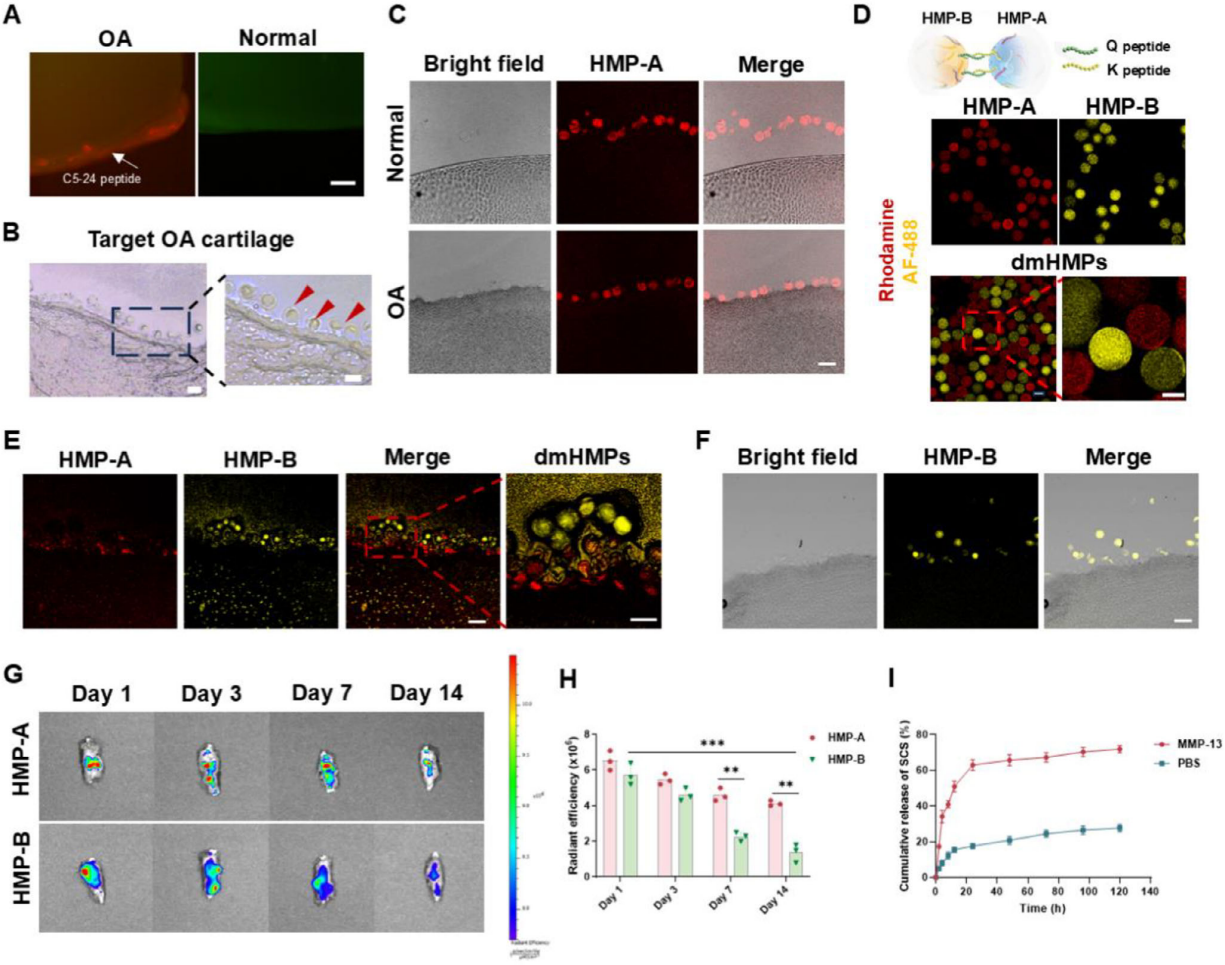
Different biological effects of HMP‐A and HMP‐B and their retention in vivo. A) Representative fluorescent image of C5‐24 peptide targeting OA cartilage. C5‐24 peptide was labeled with Rhodamine. Scale bar: 100 µm. B) Representative bright field images of HMP‐A targeting OA cartilage. Scale bar, 100 µm. C) Representative bright field, fluorescence and Merge images of HMP‐A targeting OA cartilage. HMP‐A was labeled with Rhodamine. Scale bar, 100 µm. D) Schematic and fluorescence images of HMP‐A and HMP‐B connected by annealing of K and Q peptides. HMP‐A was labeled with Rhodamine. HMP‐B was labeled with AF‐488. Scale bar, 100 µm. E) Representative fluorescence images of HMP‐A and HMP‐B connecting on the surface of ex vivo OA porcine cartilage. HMP‐A was labeled with Rhodamine. HMP‐B was labeled with AF‐488. Scale bar, 100 µm. F) Represents fluorescence image and bright field image of HMP‐B on the surface of cartilage. HMP‐B was labeled with AF‐488. Scale bar, 100 µm. G) Images of fluorescence captured from the mice joints after the injection of HMP‐A and HMP‐B for 1, 3, 7, and 14 days. HMP‐A and HMP‐B were labeled with Rhodamine. H) Quantified the fluorescence intensity of Rhodamine. I) Cumulative release curves of SCS from HMP‐B in MMP‐13 and PBS. Data are shown as means ± SD. Statistical analysis was performed using two‐way ANOVA. *P < 0.05, **P < 0.01, ***P < 0.005, and ****P < 0.001; ns, not significant.

Under OA conditions, the cartilage matrix degrades under the influence of inflammation and other factors, and the collagen in the chondrocyte matrix is exposed.^[^ ^36^ ^]^ Therefore, a polypeptide, collagen‐binding peptide C5‐24, which can accurately target the surface of OA cartilage, was screened by phage display technology.^[^ ^37^ ^]^ To investigate its binding efficiency to cartilage, Rhodamine dye was added to the C5‐24 peptide to investigate its dispersion on the surface of the cartilage. We used 0.5% trypsin to digest ex vivo porcine cartilage to simulate the OA cartilage model. Immunostainings of porcine OA cartilage demonstrated the excellent targeting performance of the C5‐24 peptide (**Figure** **3**A). Additionally, HMP‐A modified with the C5‐24 peptide displayed effective targeting and enrichment in isolated porcine OA cartilage, as observed through both bright‐field and fluorescent microscopy (Figure 3B,C), revealing a distinct targeting pattern compared to normal articular cartilage. Thus, these results demonstrate the effective targeting ability of HMP‐A toward the damaged cartilage surface in OA.

Next, we conducted an investigation to determine whether HMP‐A and HMP‐B possess the ability to autonomously assemble into dual‐modular HMP systems (dmHMPs) as anticipated, in order to achieve a synchronized regulatory mechanism for inflammation modulation and cartilage repair. K peptide was introduced to HMP‐A while Q peptide was added to HMP‐B, facilitating effective self‐assembly of both through a non‐canonical amide linkage between the K and Q peptides mediated by activated Factor XIII (FXIIIa, a naturally occurring enzyme).^[^ ^38^ ^]^ The HMP‐A and HMP‐B were labeled with distinct fluorescein to facilitate their differentiation. Immunostainings demonstrated the facile binding of HMP‐A and HMP‐B upon their co‐mixing (Figure 3D). Furthermore, we also validated the in situ binding capability of both HMPs using an ex vivo porcine OA cartilage. Immunostainings demonstrated that HMP‐B exhibited effective binding to the OA cartilage layer adjacent to HMP‐A (Figure 3E). In contrast, sole injection of HMP‐B failed to enhance enrichment within the OA cartilage layer (Figure 3F). Thus, these results suggest that HMP‐A exhibits effective binding affinity toward diseased OA cartilage and subsequently undergoes in situ assembly with HMP‐B to form multi‐effect dmHMPs.

To investigate the in vivo retention of the two HMPs, fluorescent signals of labeled HMPs in joints at 1, 3, 7, and 14 days were detected using an in vivo imaging system. Based on the fluorescence quantitative results, we observed a sustained presence of HMP‐A within the joint for 14 days (Figure 3G–H). In contrast, the signal of HMP‐B gradually declined over time and exhibited a sharp decrease at day 7. This decline in signal can be primarily attributed to the elevated expression of MMP‐13 in the OA microenvironment. The MMP‐sensitive peptide in HMP‐B undergoes cleavage, leading to disruption of the cross‐linking system and subsequent collapse of the hydrogel microsphere structure. Furthermore, the release profile demonstrated that SCS exhibited significant liberation from HMP‐B within the initial 20 h when exposed to an MMP‐13 enzyme solution, resulting in a release efficacy of 50%. In contrast, the release of SCS was comparatively sluggish in PBS with only an observed effect of 20%. Subsequently, after 100 h, the rate of release further decelerated and culminated at a final cumulative release of 75% by 120 h (Figure 3I). This differential pattern confirmed the intelligent response of HMP‐B to the pathological microenvironment associated with OA. Previous studies have indicated that the highly inflammatory microenvironment primarily affects the differentiation of SMSCs in the early stages,^[^ ^24^ ^]^ and the rapid degradation and SCS release from HMP‐B are well‐suited for this process. Conversely, the sustained presence of HMP‐A ensures long‐term recruitment and chondrogenic differentiation of SMSCs during OA treatment.

### 2.3 dmHMPs Regulated Macrophage Phenotype to Ameliorate Synovitis

**Figure 4:**
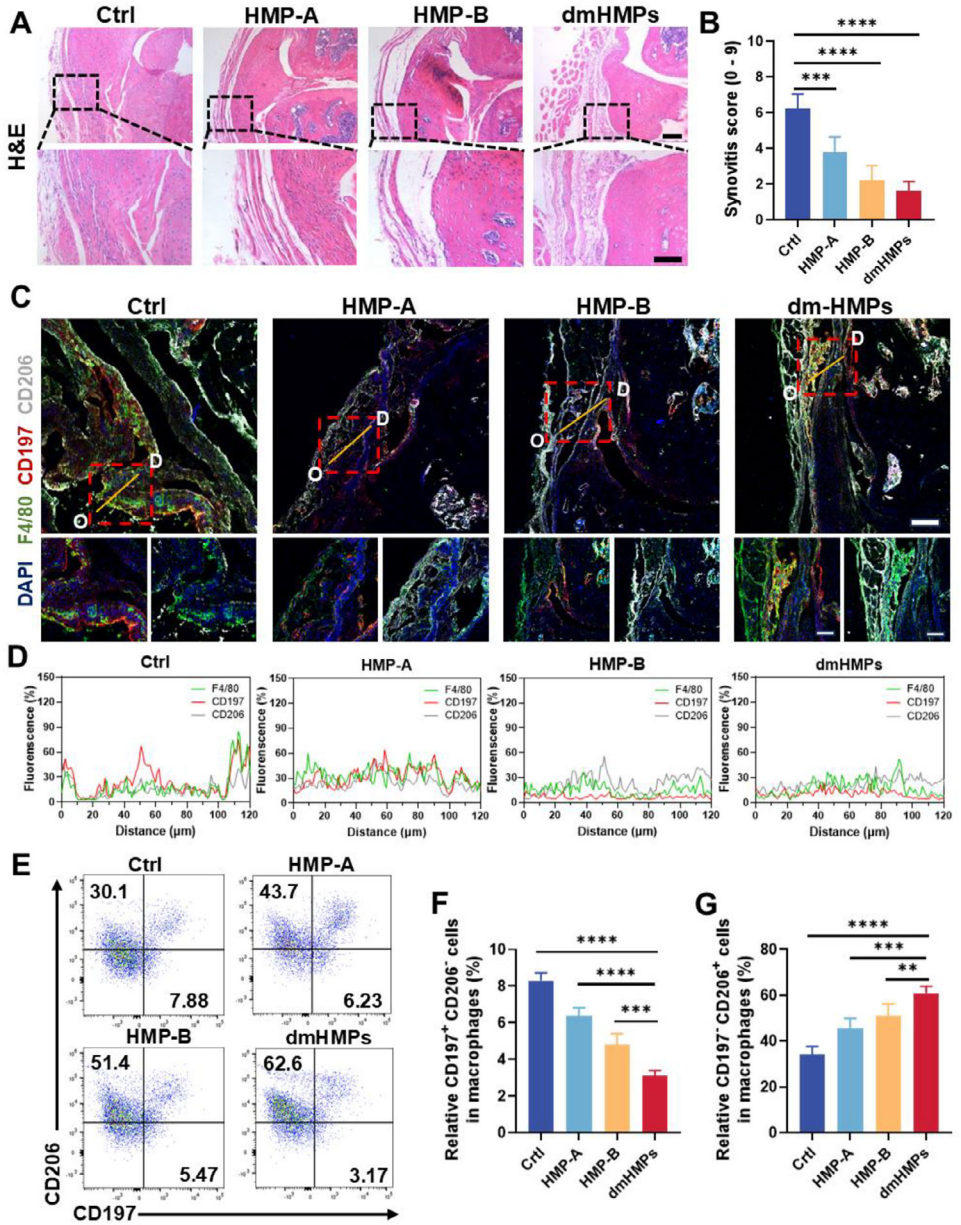
dmHMPs promote macrophage polarization in vivo and alleviate synovitis. A) H&E staining of the synovium from DMM mice after HMP‐A and HMP‐B treatment. Scale bar, 200 µm. B) Synovia scores of the synovium from DMM mice (*n *= 5). C) Representative immunostainings of F4/80, CD197, and CD206 in the synovium from DMM mice treated with HMPs for 8 weeks after OA modeling surgery. Scale bar, 200 µm. D) The fluorescence intensities of protein expression along the line segments (*O – D*) in the images. E–G) Representative flow cytometry plots E) with quantification F) of CD197^+^CD206^‐^ macrophages (M1) and G) CD197^‐^CD206^+^ macrophages (M2) polarization in Ctrl, HMP‐A, HMP‐B and dmHMPs (*n *= 4). Data are shown as means ± SD. Statistical analysis was performed using one‐way ANOVA with Tukey's post hoc test. *P < 0.05, **P < 0.01, ***P < 0.005, and ****P < 0.001; ns, not significant.

Given the frequent coexistence of synovitis in OA and its ability to activate a substantial population of pro‐inflammatory synovial macrophages, which infiltrate the injured site, secrete detrimental factors, and impede the self‐renewal and repair processes of cartilage tissue, it is imperative to effectively regulate and ameliorate the pro‐inflammatory pathological microenvironment associated with OA as a primary objective in its treatment.^[^ ^39^ ^]^ To investigate the effect of HMPs on ameliorating joint inflammation, we intra‐articular administered HMPs to mice with OA induced by destabilization of the medial meniscus (DMM) and conducted a comprehensive investigation into the extent of synovial thickening and fibrotic progression in OA restricted by HMPs. Hematoxylin and eosin (H&E) analysis that the thickening of the synovial lining layer was significantly relieved with dmHMPs compared to the Ctrl group (**Figure** **4**A). To better analyze the extent of synovitis, we evaluated the enlargement of the synovial lining cell layer, the cellular density of the synovial stroma, and the density of the inflammatory infiltrate to produce synovitis scores. The total synovitis scores in knees in the dmHMPs group were significantly lower than those in the Ctrl, HMP‐A, and HMP‐B groups (Figure 4B). Given the frequent association between synovial fibrosis and macrophage phenotypes, we subsequently examined the effect on macrophage polarization on 8 weeks after surgery. Immunostainings with quantitative analysis demonstrated that HMP‐B and dmHMPs exerted significant regulatory effects on macrophage polarization toward the M2 phenotype (Figure 4C,D), indicating that the release of SCS by HMP‐B effectively improves the inflammatory microenvironment in the joint while reducing the generation of pro‐inflammatory macrophages M1 in the synovial membrane. Flow cytometry analysis also revealed that dmHMPs significantly decreased the proportion of M1 macrophages (CD197^+^CD206^‐^ cells) and increased the proportion of M2 macrophages (CD197^‐^CD206^+^ cells) compared with Ctrl (Figure 4E–G). Together, these results suggest that dmHMPs can regulate joint inflammation through polarizing macrophages toward an M2 phenotype, thereby significantly alleviating synovitis.

### 2.4 dmHMPs Promoted SMSC‐Mediated Full‐Thickness Cartilage Repair

**Figure 5:**
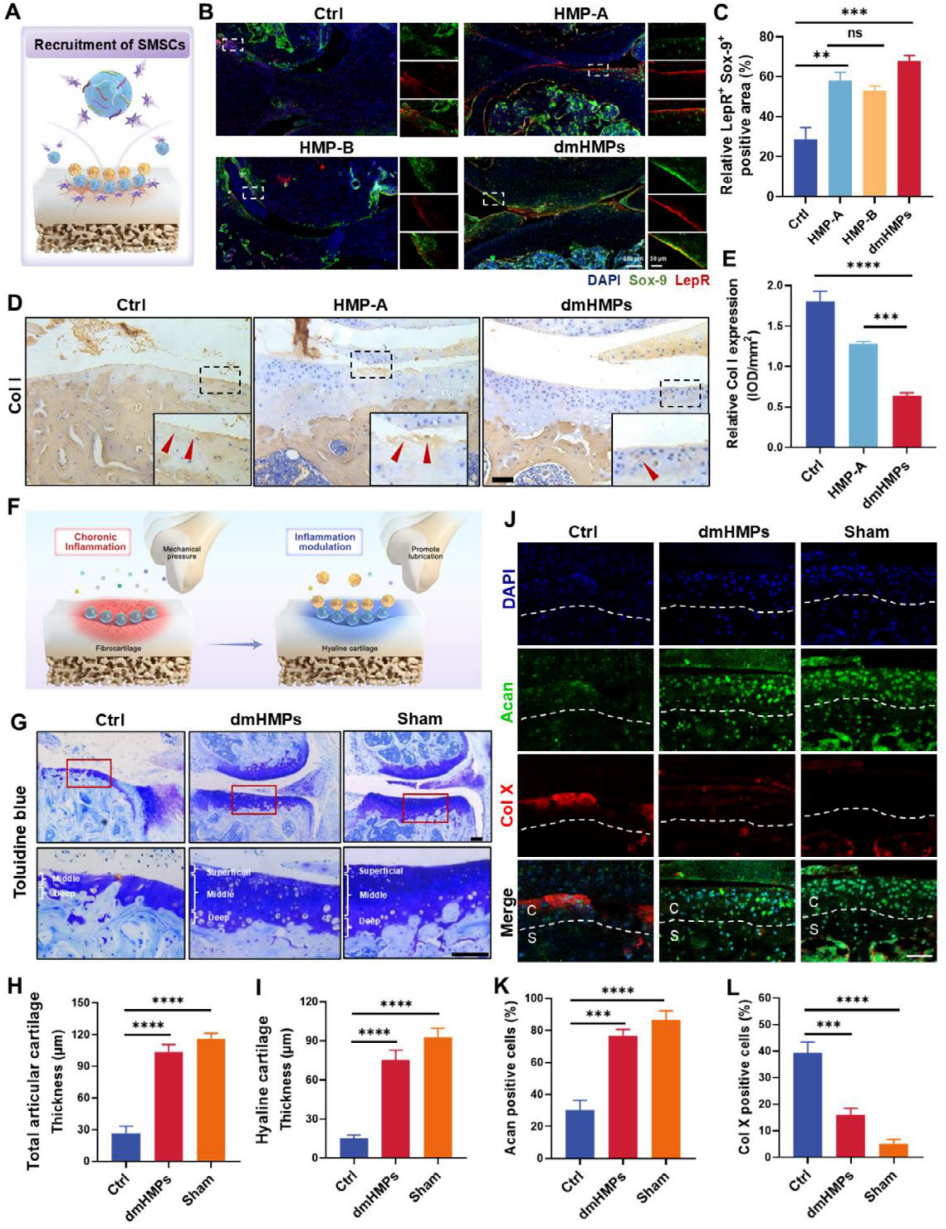
dmHMPs promote the recruitment of SMSCs to the cartilage surface and significantly enhance the formation of hyaline cartilage. A) Schematic illustration of the recruitment process of SMSCs during cartilage repair in vivo. B) Representative immunostainings of LepR and Sox‐9 in the knee joints from DMM mice treated with different treatments. The white rectangle marks the recruited SMSCs. C) Quantitative analysis of the relative LepR^+^ Sox‐9^+^ positive area in the knee joint (*n *= 5). D) Representative immunohistochemical staining for Col I of the fibrocartilage in the cartilage of DMM mice treated with HMP‐A and dmHMPs. Scale bar: 200 µm. E) Quantitative analysis of the relative Col I expression in the knee joint (*n *= 5). F) Schematic illustration of dmHMPs promotes hyaline cartilage production by promoting lubrication and modulating inflammation. G) Representative images of toluidine blue staining of Sham, Ctrl, and dmHMPs. Scale bar: 100 µm. H,I) Quantitative analysis of total articular cartilage and hyaline cartilage (*n *= 5). J) Representative immunostainings of Acan and Col‐X in the articular cartilage of Sham, Ctrl, and dmHMPs. C: Cartilage; S: Subchondral bone; White dotted line: Dividing line between cartilage and subchondral bone. Scale bar, 50 µm. K,L) Quantitative analysis of Acan positive cells and Col‐X positive cells (*n *= 5). Data are shown as means ± SD. Statistical analysis was performed using one‐way ANOVA with Tukey's post hoc test. *P < 0.05, **P < 0.01, ***P < 0.005, and ****P < 0.001; ns, not significant.

To achieve optimal treatment outcomes for OA, it is imperative to efficiently recruit SMSCs within the cartilage lesion region and regulate their downstream differentiation into chondrocytes. Therefore, we employed leptin receptors (LepR) labeling for SMSCs and Sry‐type high‐mobility‐group box‐9 (Sox‐9) labeling for chondrocytes,^[^ ^40^, ^41^ ^]^ focusing on evaluating the efficacy of dmHMPs in situ for recruiting SMSCs and facilitating their differentiation into cartilage (**Figure** **5**A). Immunostainings demonstrated that both HMP‐A and dmHMPs effectively facilitated the recruitment of LepR^+^ SMSCs in the cartilage region (Figure 5B), thereby reaffirming the efficacy of SKP peptide‐mediated recruitment of SMSCs in HMP‐A. Specifically, we observed a substantial number of regions exhibiting co‐expression of red and green fluorescence in the cartilage layer of the dmHMPs group (Figure 5B,C), indicating that surface‐enriched SMSCs on articular cartilage possess a significant propensity for chondrogenic differentiation. In contrast, while HMP‐A could also efficiently recruit LepR^+^ SMSCs, it lacked the ability to regulate their differentiation into chondrocytes. HMP‐A was capable of effectively recruiting SMSCs to the injured cartilage area; however, its inability to adequately modulate the immune microenvironment hindered the repair process. We thus further assessed the extent of fibrocartilage production following the treatment with various HMPs. Immunohistochemical staining for Col I revealed severe articular cartilage wear in both the Ctrl and HMP‐A groups, leading to a significant reduction in surface hyaline cartilage and an elevated expression of fibrocartilage rich in Col I (Figure 5D,E). In contrast, dmHMPs exhibited superior ability to preserve articular cartilage integrity and suppress the expression of Col I, thereby mitigating fibrocartilage formation. This limitation primarily stemmed from the influence of the inflammatory microenvironment in OA, where recruited SMSCs often differentiated uncontrollably into fibrous tissues, leading to repair failure (Figure 5F).

dmHMPs can not only inhibit the formation of articular fibrocartilage but also promote the regeneration of full‐thickness articular cartilage. It is important to note that articular cartilage is spatially organized into three distinct zones: the superficial zone, middle zone, and deep zone.^[^ ^42^ ^]^ Among these zones, the superficial zone plays a pivotal role in joint function by secreting lubricin to enhance lubrication and by resisting shear stress on the articular cartilage surface, thereby maintaining cartilage homeostasis.^[^ ^43^, ^44^ ^]^ Through toluidine blue staining observation, it can be found that the thickness comparison of the superficial cartilage and the hypertrophic cartilage shows that the superficial hyaline cartilage in the Ctrl group is severely eroded and almost loses its lubrication function (Figure 5G). Through quantitative analysis of total articular cartilage and hyaline cartilage, we found that dmHMPs effectively restored full‐thickness cartilage (Figure 5H,I). Immunofluorescence staining indicated that the Aggrecan (Acan) in the superficial cartilage of the DMM group was significantly reduced, with Acan‐positive cells accounting for only ≈20%, while the expression of Col X was significantly increased, especially in the superficial cartilage, suggesting chondrocyte hypertrophy and apoptosis, thereby losing the morphology of the superficial cartilage (Figure 5J). dmHMPs could effectively alleviate the degradation of Acan, with Acan‐positive cells accounting for ≈80%, while Col X‐positive cells accounted for ≈20%, which was significantly downregulated compared with the DMM group (Figure 5K,L). These results suggest that the integrity of articular cartilage, especially hyaline cartilage, can be restored through the regulation of inflammation and effective cartilage repair mediated by SMSCs.

**Figure 6:**
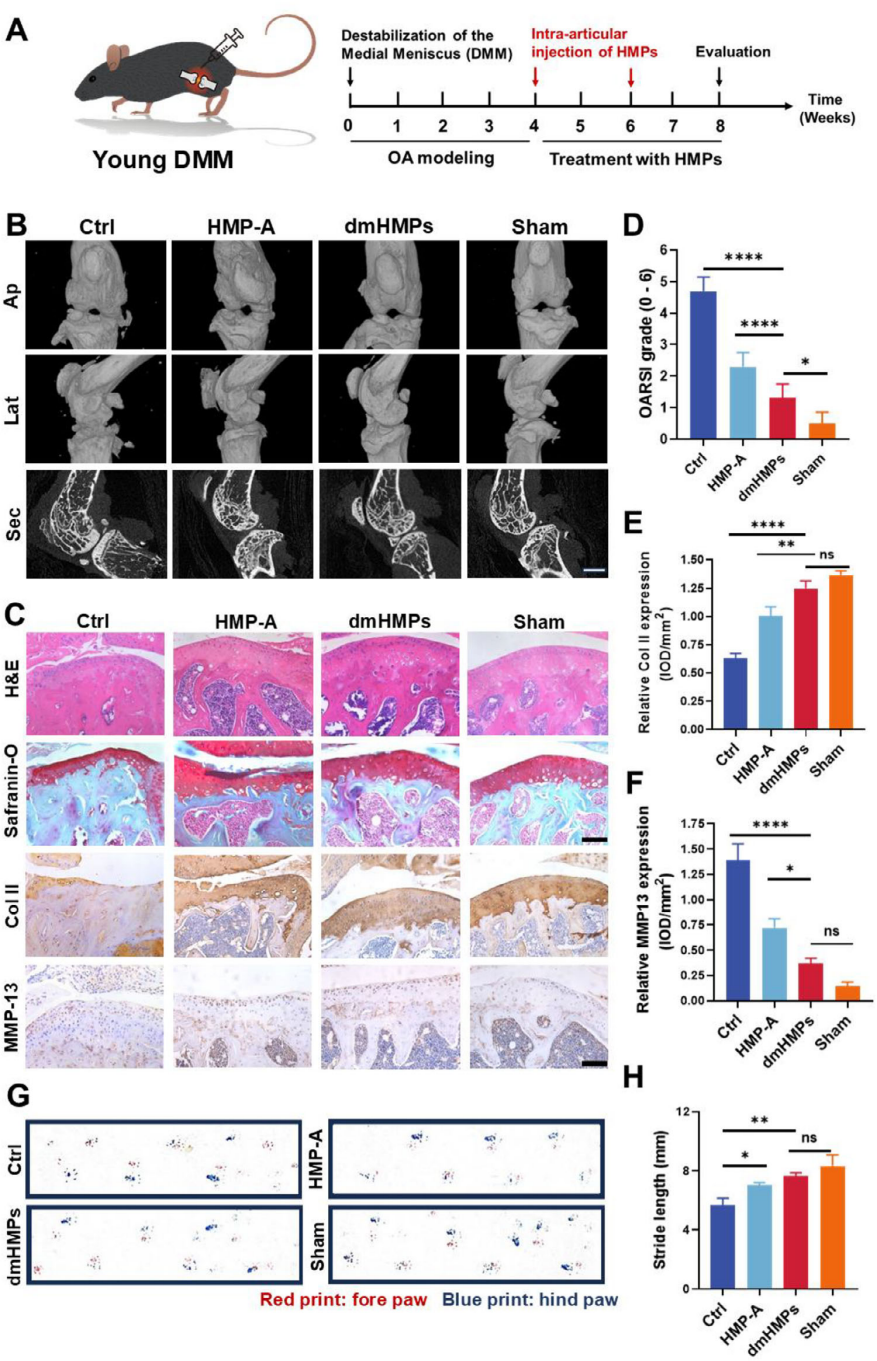
dmHMPs alleviate cartilage degeneration in young OA mice after DMM surgery. A) Schematic illustration of the experimental design of DMM modeling and intraarticular injection in young mice (3 months old). B) Representative Micro‐CT images in anterior‐posterior (AP), lateral (LAT), and section view of the young mice knee joints. C) Representative images of H&E staining, Safranin O‐fast green staining, and immunohistochemical staining for Col2a1, and MMP‐13 of the young OA mice. Scale bar:200 µm. D) OARSI grades of mice joints in each group (*n *= 5). E,F) Quantification of relative expression of Col II and MMP‐13 in cartilage (*n *= 5). G) Gait analysis for DMM mice in each group. Red print: fore paw; blue print: hind paw. H) Quantification of stride length (*n* = 5). Data are shown as means ± SD. Statistical analysis was performed using one‐way ANOVA with Tukey's post hoc test. *P < 0.05, **P < 0.01, ***P < 0.005, and ****P < 0.001; ns, not significant.

Next, to further evaluate the therapeutic efficacy of intra‐articular injection of dmHMPs on OA repair, a series of comprehensive assessments were conducted. The experimental timeline is presented in **Figure** **6**A. Prior in vivo fluorescence retention studies demonstrated that the fluorescence signals of HMP‐A and HMP‐B diminished considerably after two weeks (Figure 3G,H), indicative of substantial degradation. To optimize therapeutic efficacy, we have determined a two‐week injection interval for the administration of dmHMPs in the treatment of OA. Osteophytes serve as crucial diagnostic indicators in the identification of OA. They arise from subchondral osteosclerosis and secondary bone hyperplasia, resulting from an inflammatory response triggered by cartilage injury, thereby exacerbating joint pain associated with OA. Micro‐computed tomography (Micro‐CT) analysis revealed an elevated presence of periarticular osteophytes in the Ctrl group, indicating dysregulated bone remodeling (Figure 6B). In contrast, the dmHMPs group exhibited minimal knee osteophytes comparable to those observed in the Sham group (Figure 6B). Furthermore, the cross‐sectional analysis demonstrated that the subchondral bone in the dmHMPs group displayed enhanced stability and absence of subchondral osteosclerosis, resembling normal subchondral bone tissue more closely than other groups.

To investigate the recovery of damaged articular cartilage in OA mice following intra‐articular injection of various HMPs, we performed histological analysis H&E as well as Safranin O/Fast Green staining. Histological analysis revealed that the knee joints in the dmHMPs group exhibited preserved cartilage integrity and showed minimal signs of degeneration at 8 weeks (Figure 6C). In addition, the Osteoarthritis Research Society International (OARSI) score of the dmHMPs group was significantly lower than that of the Ctrl and HMP‐A groups and was similar to that of the Sham group (Figure 6D). The expression of the synthetic cartilage‐related protein Col II and the degrading enzyme MMP‐13 on the cartilage surface of OA mice were also assessed through immunohistochemical staining. The results revealed a significant upregulation in Col II expression in the dmHMPs group compared to both Ctrl and HMP‐A groups, while no significant difference was observed when compared to the Sham group (Figure 6C,E). Additionally, treatment with dmHMPs significantly reduced MMP‐13 expression on the cartilage surface (Figure 6C,F), indicating that it effectively alleviated the degradation of the cartilage matrix in OA through inflammation regulation.

Next, to evaluate the recovery of joint motor function, we conducted a gait analysis to assess in OA mice. The claudication of mice was assessed by analyzing the separation of the paw prints as well as changes in stride length. The results revealed a significant convergence in the prints of both front and hind paws following dmHMPs treatment, accompanied by step length and stride length resembling those observed in the Sham group (Figure 6G,H), indicating a substantial alleviation of claudication due to dmHMPs treatment. Together, these results suggest that dmHMPs facilitate the recruitment of SMSCs to the damaged articular cartilage layer and promote their differentiation into chondrocytes, thereby enhancing the therapeutic potential for OA.

### 2.5 Differential mRNA Expression Reveals dmHMPs Therapeutic Mechanism

**Figure 7:**
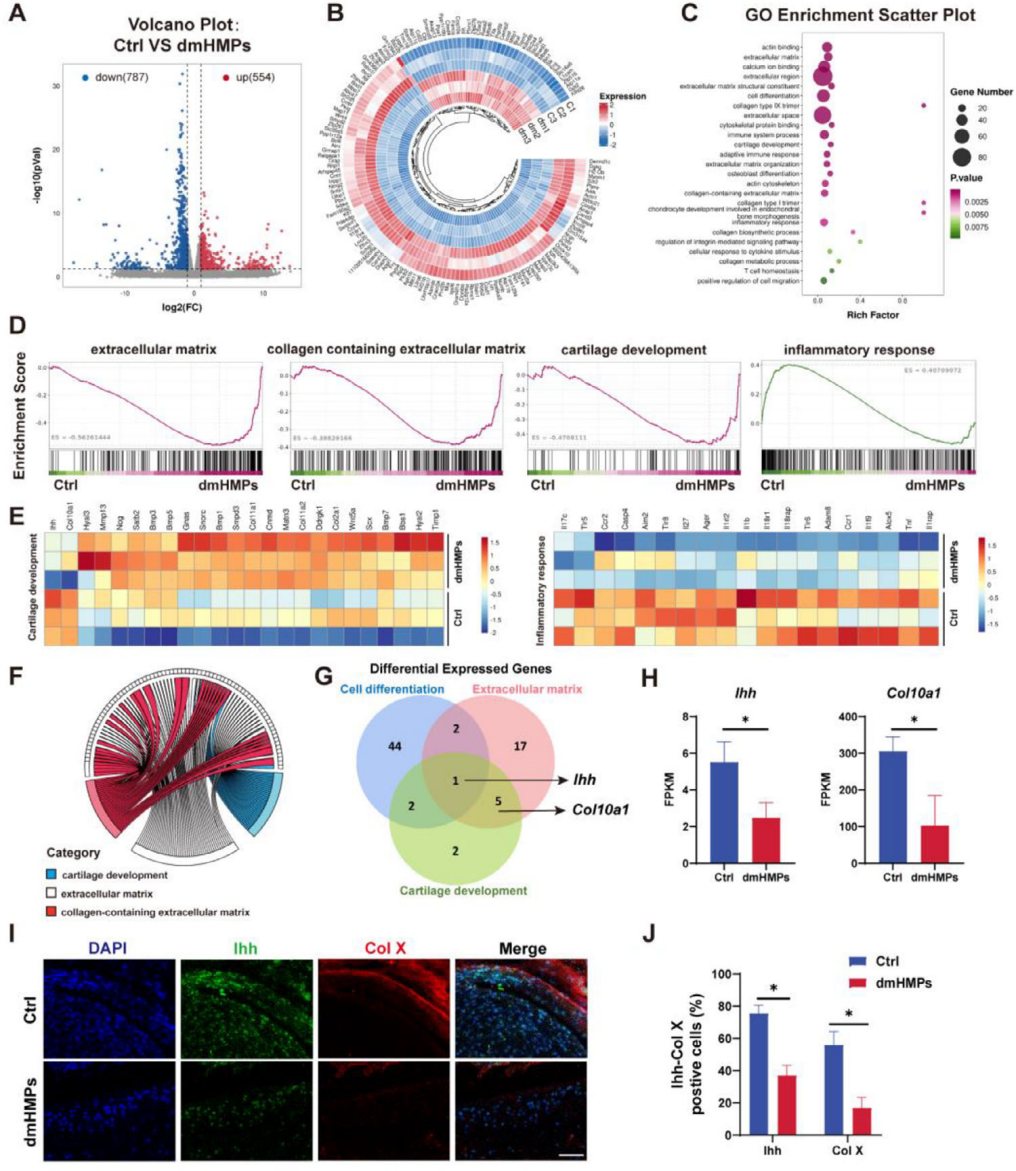
Intrinsic mechanisms of dmHMPs promoting chondrogenesis and alleviating OA progress in vivo. A) Volcano plot of transcriptomic analysis of differentially expressed mRNA between Ctrl and dmHMPs (*p *< 0.05, |log_2_(fold change)| > 1). B) Heat map representing the top 100 significant gene expression values between Ctrl and dmHMPs. C) Representative GO enrichment pathways of significantly differentially expressed genes (DEGs) between Ctrl and dmHMPs. D) GSEA enrichment analysis of extracellular matrix, collagen‐containing extracellular matrix, cartilage development, and inflammatory response between Ctrl and dmHMPs. E) Heatmaps of genes related to cartilage development and inflammatory response (*n* = 3). F) Expression and crossover of each gene of extracellular matrix, collagen‐containing extracellular matrix, and cartilage development. G) Venn diagram of differentially expressed genes (DEGs) in cell differentiation, extracellular matrix, and cartilage development between the Ctrl and dmHMPs groups. H) FPKM values of *Ihh* and *Col10a1* in mRNA sequencing. Scale bar: 100 µm. I) Representative immunostainings of Ihh and Col‐X in the articular cartilage of Ctrl and dmHMPs. J) Quantitative analysis of Ihh positive cells and Col‐X positive cells (*n *= 5). Data are shown as means ± SD. Statistical analysis was performed using two‐tailed Student's *t*‐tests and one‐way ANOVA with Tukey's post hoc test. *P < 0.05, **P < 0.01, ***P < 0.005, and ****P < 0.001; ns, not significant.

To obtain insight into the mechanism by which dmHMPs alleviate cartilage degeneration of young OA mice, we performed mRNA sequencing to analyze the effect of dmHMPs treatment for 2 weeks after OA modeling. Volcano plot analysis of differentially expressed genes (DEGs) was performed (**Figure** **7**A). Compared with the Ctrl group, a total of 1341 DEGs were identified in the dmHMPs group, of which 554 DEGs were significantly up‐regulated and 787 DEGs significantly down‐regulated were identified. The circular heat map of cluster analysis analyzed the top 100 differential genes and directly demonstrated the significant changes of genes between the Ctrl group and the dmHMPs group (Figure 7B). Furthermore, we analyzed differentially expressed genes through enriched gene ontology (GO). GO enrichment scatter plot revealed that DEGs between Ctrl and dmHMPs are mainly enriched in extracellular matrix, collagen‐containing extracellular matrix, cartilage development, inflammatory response, and other signaling pathways, which are closely related to the regulation of OA (Figure 7C). Gene‐set enrichment analysis (GSEA) demonstrated the regulatory role of dmHMPs in the above four pathways compared with the Ctrl group. dmHMPs were beneficial to the production of extracellular matrix (ECM), especially collagen‐containing ECM, and regulated the inflammatory response to promote cartilage development (Figure 7D). The analysis of differentially expressed genes in the cartilage development and inflammatory response pathways revealed a significant upregulation of *Sox9* and *Col‐2a1*, which are associated with extracellular matrix regulation in cartilage. Meanwhile, there was a significant downregulation of inflammatory genes including *Il‐1b*, *Ccr2*, *Adamts5*, and *Tnf* in the inflammatory response pathway (Figure 7E). In addition, the intersection analysis of the extracellular matrix, collagen‐containing extracellular matrix, and cartilage development showed that dmHMPs promoted cartilage development through the improvement of the extracellular matrix (Figure 7F). The analysis of the results from the Venn diagram reveals that the gene closely related to cartilage development, cell differentiation, and extracellular matrix is the Indian hedgehog (*Ihh*) (Figure 7G). As a marker of hypertrophic cartilage, *Ihh* is not expressed in healthy articular cartilage, but its expression is significantly elevated in OA cartilage (Figure 7H). Abnormal expression of *Ihh* in articular cartilage can lead to abnormal cartilage matrix remodeling and mineralization, and further aggravate cartilage injury. To ensure that regenerated cartilage can function for a long time, hypertrophy of chondrocytes must be inhibited. In addition, the hypertrophic cartilage marker Col X mentioned earlier was also significantly increased (Figure 7I). The expression levels of the two genes were assessed using immunofluorescence staining, and the findings were in agreement with the sequencing data. Compared to the dmHMPs group, the Ctrl group exhibited a significant upregulation in the expression of Ihh and Col X. These results indicate that the dual‐module structured dmHMPs can effectively reduce hypertrophy and apoptosis of articular cartilage.

Collectively, dmHMPs significantly enhanced the synthesis of cartilage extracellular matrix, particularly collagen, in mice with OA and effectively modulated the immune microenvironment in vivo. At the same time, dmHMPs can effectively reduce the expression of cartilage hypertrophy markers Ihh and Col10a1, thereby reducing cartilage hypertrophy and extracellular matrix degradation. Consequently, treatment with dmHMPs exhibited remarkable benefits for promoting cartilage development and alleviating OA.

### 2.6 dmHMPs Alleviated the Progression of OA in Aged Mice

**Figure 8:**
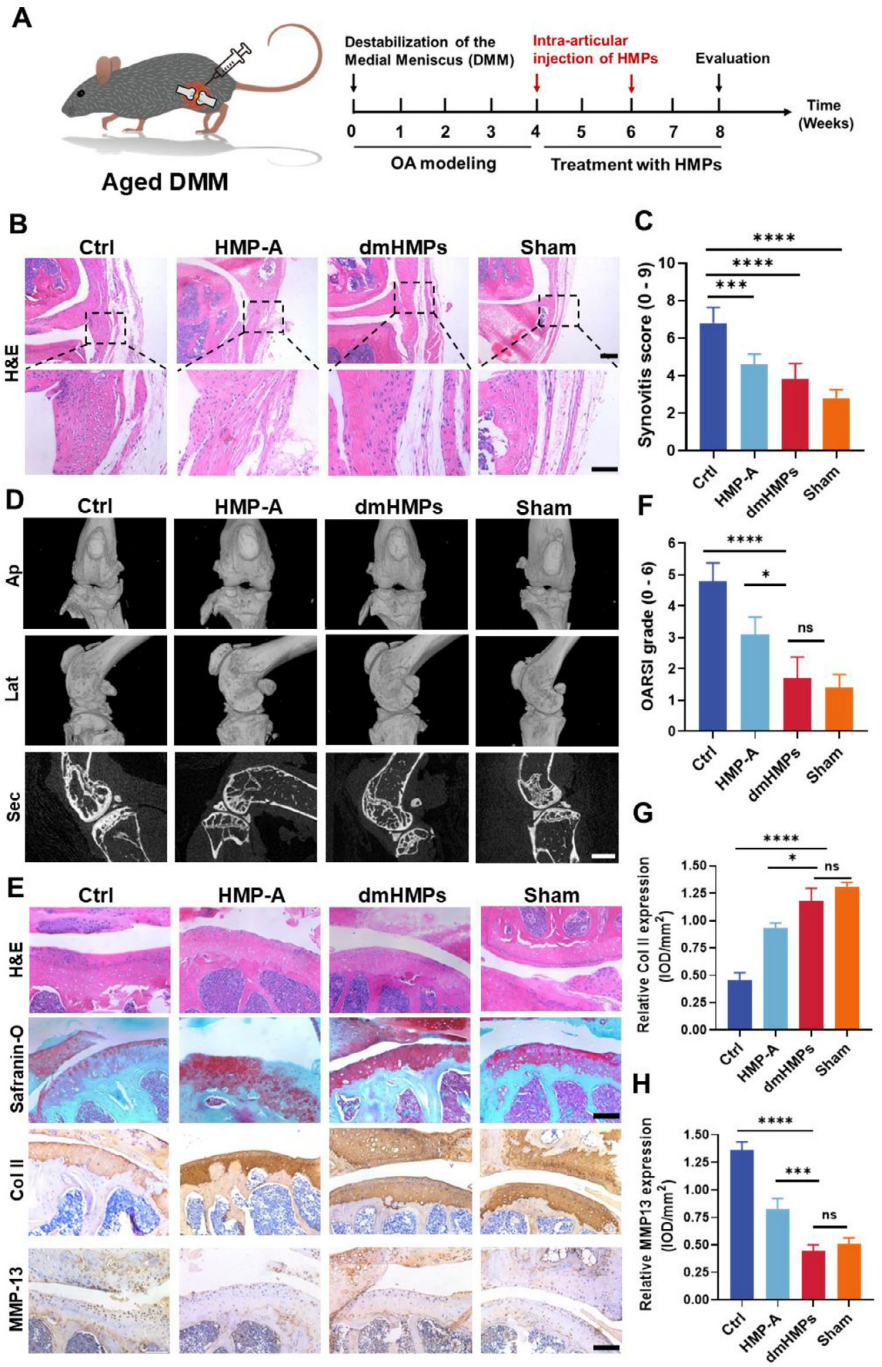
dmHMPs alleviate the progression of OA in aged mice. A) Schematic illustration of the experimental design of DMM modeling and intra‐articular injection in aged mice (18 months old). B) Representative H&E staining of the synovium from aged DMM mice in each group. C) Synovia scores of the synovium from aged DMM mice (*n* = 5). D) Representative Micro‐CT images in anterior–posterior (AP), lateral (LAT), and section view of the young mice knee joints. E) Representative images of H&E staining, Safranin O‐fast green staining, and immunohistochemical staining for Col II, and MMP‐13 of the aged OA mice. Scale bar: 200 µm. F) OARSI grades of mice joints in each group (*n* = 5). G,H) Quantification of relative expression of Col II and MMP‐13 in cartilage (*n *= 5). Data are shown as means ± SD. Statistical analysis was performed using one‐way ANOVA with Tukey's post hoc test. *P < 0.05, **P < 0.01, ***P < 0.005, and ****P < 0.001; ns, not significant.

Aging is a prominent risk factor in the development and progression of OA. Specifically, the heightened expression of senescence‐associated secretory phenotype within the aging microenvironment, compromised cellular proliferation and metabolism, as well as diminished differentiation potential of stem cells collectively impede clinical interventions aimed at repairing OA in an aged population.^[^ ^45^ ^]^ To further assess the efficacy of dmHMPs in treating OA within complex aging microenvironments, we established a DMM model using 18‐month‐old male mice. The experimental timeline is depicted in **Figure** **8**A. Synovitis serves as a pivotal indicator of inflammation in OA, particularly in aged models, thus prompting an investigation into the impact of dmHMPs on the regulation of inflammatory processes through synovitis assessment. H&E analysis revealed that dmHMPs exhibited a milder degree of synovitis and reduced fibrosis compared to the Ctrl and HMP‐A groups (Figure 8B). Additionally, the synovitis score in the dmHMPs group was significantly lower than that in the Ctrl and HMP‐A groups (Figure 8C). Similarly, the therapeutic effect of HMPs on aged mice was investigated using Micro‐CT analysis, histological staining (H&E and Safranin‐O/Fast Green), and immunohistochemical staining (Col II and MMP‐13) Micro‐CT analysis showed that compared to the Ctrl group, treatment with dmHMPs significantly attenuated osteophyte formation around the joint and preserved the stability of subchondral bone. Moreover, the administration of dmHMPs effectively maintained the structural integrity and morphology of the joint, resembling those observed in the Sham group (Figure 8D).

Additionally, H&E and Safranin O/Fast Green staining demonstrated that dmHMPs effectively preserved cartilage integrity and significantly mitigated the loss of cartilage extracellular matrix (Figure 8E). Moreover, the OARSI score in the dmHMPs group was markedly lower compared to both the Ctrl and HMP‐A groups (Figure 8F). Immunohistochemical analysis showed that dmHMPs could maintain the expression level of Col II in cartilage, which was significantly higher than Ctrl and HMP‐A, while also substantially reducing the expression of MMP‐13 (Figure 8G,H). Together, these findings suggest that the effective ability of dmHMPs to ameliorate cartilage degradation and retard age‐related OA progression, with therapeutic efficacy comparable to that observed in young mice.

## 3 Discussion

OA is a predominant degenerative joint disease and a leading cause of disability among the elderly.^[^ ^46^ ^]^ Despite extensive research in recent decades aimed at developing biomaterials for cartilage repair, many strategies have not successfully translated into clinical practice^[^ ^8^ ^]^ This often leads to the formation of fibrocartilage tissue post‐implantation, which inadequately meets the mechanical demands of healthy cartilage.^[^ ^16^, ^17^ ^]^ In this study, we designed in situ self‐assembled dmHMPs to protect articular cartilage and facilitate the repair of damaged tissue through synchronized regulatory mechanisms. These dmHMPs effectively orchestrated a dynamic interplay among inflammation microenvironment modulation, recruitment of SMSCs, and their differentiation during the repair process. Our findings demonstrate a biomaterial construction strategy that achieves in situ assembly and responds to the pathological microenvironment, enabling spatiotemporal regulation of OA. This approach offers promising avenues for future therapeutic interventions.

A typical feature of the early immune response in OA is the presence of large numbers of immune cells in the synovium, with the most abundant being synovial macrophages, which orchestrate the inflammatory and resolution phases after tissue injury.^[^ ^12^, ^20^ ^]^ However, an excessive pro‐inflammatory macrophage‐mediated inflammatory response can impede the subsequent cascade of SMSCs‐mediated cartilage repair. Consequently, disruption of pro‐inflammatory macrophage infiltration into the synovium is a potential therapeutic approach. dmHMPs release SCS in response to the inflammatory environment in the joints through the MMP‐13‐sensitive peptide for immune regulation.^[^ ^47^, ^48^ ^]^ In our previous studies, SCS has been demonstrated to promote the polarization of macrophages from pro‐inflammatory M1 phenotype to pro‐regeneration M2 phenotype via the IL‐4‐mediated Stat‐6 signaling pathway.^[^ ^27^ ^]^ Therefore, immunostainings of synovium and in vivo flow cytometry analysis demonstrated that dmHMPs effectively reduce the proportion of M1 macrophages, thereby alleviating synovial inflammation. Moreover, the lubricating performance of HMP‐B loaded with SCS is significantly enhanced. This improvement can be attributed to the structural similarity between SCS and the natural lubricant chondroitin sulfate, both of which possess abundant sulfonic acid groups, thereby further enhancing the lubricating properties of HMP‐B.^[^ ^49^ ^]^ The immunohistochemistry staining shows that dmHMPs reduce the expression of Col I, a fibrocartilage marker, by regulating inflammation and lubrication performance, proving that dmHMPs promote the dynamic coupling between inflammation regulation and chondrogenic differentiation of SMSCs, which is beneficial for solving the problem of intrinsic cartilage damage self‐repair in vivo.

Due to a very close embryologic link between the cartilage and the synovium, SMSCs have been shown to enhance cartilage repair, making them a potential therapeutic option for superficial injuries in OA.^[^ ^50^, ^51^ ^]^ However, the migration of SMSCs with high chondrogenic potential from the synovial lining to the damaged cartilage surface is hindered by OA‐induced inflammation and mechanical instability. Consequently, the failure to promptly repair the superficial cartilage leads to an acceleration of cartilage degeneration, thus perpetuating a detrimental cycle. Therefore, promoting the migration of SMSCs to the damaged cartilage surface is a potential therapeutic approach for OA cartilage repair. By emulating the structural arrangement of bone marrow homing peptide, a concise peptide sequence SKPPGTSS (SKP) has been documented to facilitate MSC homing,^[^ ^52^, ^53^ ^]^ and it has been confirmed to be beneficial for SMSCs to recruit to the superficial cartilage in aged OA mice.^[^ ^22^ ^]^ Therefore, SKP peptide was added to dmHMPs, and through in vitro co‐culture, it was found that HMP‐A loaded with SKP could selectively recruit SMSCs. In vivo, immunofluorescence images showed that SMSCs were significantly enriched in the damaged cartilage area and highly expressed in Sox‐9, a chondrogenic transcription factor. This indicates that dmHMPs can effectively recruit SMSCs and induce chondrogenesis. The 3D culture of dmHMPs simulates the growth environment of cells in vivo and provides more cell‐to‐cell contact and interaction with ECM, thereby promoting the differentiation of SMSCs toward chondrocytes. Studies have shown that compared with 2D culture, MSCs can express chondroblast genes more effectively and have higher levels of ECM expression, such as collagen II (Col II) and aggrecan (Acan).^[^ ^54^ ^]^ Our findings substantiate these observations, as RNA sequencing analyses demonstrate that treatment with dmHMPs markedly enhances ECM production, particularly in collagen‐associated matrices, thereby promoting cartilage development. Furthermore, this study elucidates a critical mechanism underlying this process: the regulation of chondrogenic differentiation by dmHMPs is predominantly mediated via integrin α5β1. This integrin functions as a pivotal receptor that, upon activation, initiates downstream signaling pathways responsible for synthesizing cartilage‐specific ECM components.

The pathological process of OA is always accompanied by significant degeneration of cartilage. Full thickness regeneration of articular cartilage is essential for the treatment of osteoarthritis.^[^ ^55^ ^]^ Once the cartilage has undergone pathological changes, such as wear, thinning, or absence, the normal biomechanical and motor function of the joint will be affected. In this study, dmHMPs can effectively prevent the degradation of ECM Acan and Col II through the regulation of inflammation, and restore the spatial architecture of articular cartilage. Moreover, mRNA sequencing and immunofluorescence staining demonstrated that dmHMPs significantly reduced the expression of Ihh and Col10a1, which are markers of chondrocyte hypertrophy. Insights from previous research have shown that heparin, a highly sulfated glycosaminoglycan (GAG), can effectively inhibit the activity of the Hedgehog (HH) signaling pathway, thereby reducing cartilage hypertrophy and guiding MSCs to form non‐mineralized or non‐hypertrophic cartilage tissue in vivo, and this research ultimately identified GAG sulfation as a crucial niche instruction signal to determine the chondral stem‐cell fate via silencing of prohypertrophic pathways.^[^ ^56^ ^]^ Therefore, SCS, as a sulfated polysaccharide with a heparin‐like structure, has similar biological functions as heparin to effectively silence hypertrophy signals. Finally, dmHMPs loaded with SCS can effectively reduce cartilage hypertrophy, inhibit the expression of Ihh and Col X, and mediate the differentiation of SMSCs into chondrocytes.

## 4 Conclusion

In summary, this study recognizes the interdependence of immune regulation and cartilage regeneration, leading to the development of dmHMPs that can adaptively respond to the evolving needs from immune modulation to the recruitment of SMSCs for repairing damaged cartilage. The dmHMPs dynamically couple in vivo to achieve balanced regulation of inflammation and chondrogenic differentiation of SMSCs. Moreover, these particles effectively inhibit hypertrophy and mineralization of articular cartilage, significantly promoting full‐thickness cartilage regeneration. This multifaceted approach addresses critical challenges associated with cartilage regeneration in OA. Beyond immediate therapeutic applications, this study's implications suggest future research should focus on optimizing the formulation and delivery mechanisms of dmHMPs to enhance their efficacy and specificity across various stages of OA. Additionally, exploring the combinatory effects of dmHMPs with other therapeutic modalities, such as growth factors or gene therapy, could further improve outcomes in cartilage repair. Ultimately, translating these findings into clinical practice may provide a novel strategy for restoring joint function and improving the quality of life for patients suffering from OA.

## 5 Experimental Section

### HAMA Synthesis

The synthesis of HAMA was based on the previously researched method.^[^ ^57^ ^]^ Briefly, 5 g hyaluronic acid (MW = 60 kDa, Bloomage Freda Biopharm Co., Ltd., China) was dissolved in 250 mL deionized water in a three‐necked round‐bottomed flask with a magnetic stirring rod. Stir vigorously at room temperature for 20 min to promote complete dissolution of HA. While stirring, the pH of the HA solution was adjusted to 8.5 using 1 m NaOH. Subsequently, 11.25 mL of methacrylic anhydride (MA, Aladdin) was added dropwise while continuously monitoring and readjusting the pH to 8.5 as necessary throughout the addition process. The reaction would be opaque and white because of the emulsion of methacrylic anhydride. The reaction was allowed to proceed on ice for 4 h while maintaining the pH at 7.5–8.5. All products were dialyzed for a week, the water was changed once a day, and finally freeze‐dried.

### Preparation of Hydrogel Microparticles

The production of hydrogel microparticles (HMP) was achieved through the utilization of microfluidic techniques. The droplets were generated utilizing a microfluidic system for water‐in‐oil segmentation. In this study, two different hydrogel microparticles, namely HMP‐A and HMP‐B were prepared. For the preparation of HMP‐A, the aqueous phase was a mixture of 5 wt.% HAMA with 500 µm C5‐24 peptide (Ac‐DLQYWYPIWDTHC‐NH_2_, Genscript), 500 µm K‐peptide (Ac‐FKGGERCGNH_2_, Genscript), and 10 mm SKP‐peptide (Ac‐SKPPGTSSC‐NH_2_, Genscript). For the preparation of HMP‐B, the aqueous phase was a mixture of 5 wt.% HAMA with 8 mm matrix metalloproteinase 13 (MMP‐13) sensitive peptide (Ac‐GCPLGMRGC‐NH_2_, Genscript), 500 µm Q‐peptide (Ac‐NQEQVSPLGGERCG‐NH_2_, GenScript), and 1 wt.% Sulfated Chitosan (SCS). The oil phase was paraffin oil with 5% Span80. Finally, the aqueous phase and the oil phase were independently infused into inlets of a cross‐shear microfluidic device to form the hydrogel droplets by shear forces and hydrophobic interactions at intersection points. The collected droplets cross‐linked at room temperature for one day by a Michael addition reaction. The hydrogel microparticles were ultimately obtained after complete crosslinking. After that, the collected HMPs were rinsed with ether and deionized water for three times to wash off excess oil and other additives. Fluorescent hydrogel microparticles were prepared by adding 10 µm Rhodamine to the aqueous phase of HMP‐A and 10 µm AlexaFluor‐488 to the aqueous phase of HMP‐B in the above preparation process. The preparation of fluorescent microparticles was advantageous for in vivo and in vitro observations.

### Characterizations of Hydrogel Microparticles

The optical morphology and diameter of droplets and hydrogel microparticles were observed using an inverted optical microscope (DMI‐8, Leica, Germany). The surface morphology and microstructure of lyophilized HMPs were observed by scanning electron microscopy (SEM). The sample was observed under a scanning electron microscope (S‐3400, Hitachi, Japan) after gold‐spraying treatment. The composition of HMPs and the distribution of elements were measured by EDS.

### Tribological Tests

The tribological tests were conducted using high‐frequency reciprocating friction and wear tester under reciprocating sliding conditions (UMT‐2, Bruker, Germany). In order to simulate real cartilage friction in the body, porcine cartilage was used for testing. The tribological test was carried out to prepare OA cartilage by soaking porcine cartilage with 0.5% trypsin. OA cartilage pieces with flat surfaces were selected to be fixed on the friction and wear test machine, and then a reciprocating lubrication test was conducted. According to previous studies, to simulate the frictional force experienced by human knee cartilage, the normal force per cm^2^ of cartilage was ≈5 to 10 N.^[^ ^58^, ^59^ ^]^ Therefore, in this study, normal forces of 5 and 10 N were applied to porcine cartilage to simulate the frictional force experienced by human joints. The specific test parameters were as follows: friction ball (8 mm PTFE friction ball), load (5, 10 N), amplitude 4 mm, frequency (1 Hz), friction time (15 min). OA cartilage was placed in 20 mg mL^−1^ HMP‐A and HMP‐B solutions, then the cartilage was fixed on the friction platform and the hydrogel microparticle solutions were poured into the sample pool for the tribological test. The friction coefficient of each group of samples under 5 and 10 N loads was measured, respectively. Each group of samples was measured three times.

### Cartilage Targeting Performance of Hydrogel Microparticles

First, porcine cartilage was used to simulate the ex vivo OA model. The specific procedures were as follows: Healthy porcine articular cartilage was digested with 0.5% trypsin at 37 °C for 3 h, HBSS buffer was used to wash after digestion for 5 min, and 20% fetal bovine serum was used to terminate digestion for 10 min. HMP‐A contains collagen‐targeting peptide C5‐24, and C5‐24 has good targeting performance. In vitro experiments, OA porcine articular cartilage (5 mm in diameter and 42 mm in thickness) was immersed in C5‐24 peptide solution grafted with Rhodamine fluorescence for 1 h, and then the fluorescence intensity of porcine cartilage was observed by laser confocal microscope (Leica TCS SP8). In addition, OA porcine articular cartilage and normal porcine articular cartilage were immersed in HMP‐A solution for 1 h, then removed, and then frozen and sliced to observe the targeting performance of HMP‐A on articular cartilage by optical microscope and laser confocal microscope, respectively.

### Annealed Connection of HMP‐A and HMP‐B

For every 50 µL of dried HMP‐A and HMP‐B, 2 µL of Factor XIII (250 U mL^−1^) and 1 µL of thrombin (200 U mL^−1^ in 200 mm Tris‐HCl, 150 mm NaCl, and 20 mm CaCl_2_) was added and mixed via thorough pipetting. A 30 min incubation time at 37 °C was required to form a connection of HMP‐A and HMP‐B. The connection between the grafted fluorescence HMP‐A and HMP‐B was observed by a laser confocal microscope. In addition, the annealed connection of HMP‐A and HMP‐B was investigated by using ex vivo OA porcine cartilage. First, OA cartilage was soaked in HMP‐A solution for 2 h, and then OA porcine cartilage was soaked in HMP‐B solution for 2 h, and a solution of factor XIII (250 U mL^−1^) and 1 µL of thrombin (250 U mL^−1^ in 200 mm Tris‐HCl, 150 mm NaCl, and 20 mm CaCl_2_) was added at the same time. A 30 min incubation time at 37 °C was required to form a connection of HMP‐A and HMP‐B. Finally, the samples were frozen and sliced, and observed by laser confocal microscope.

### In Vivo Retention of Hydrogel Microparticles in Joint

Twenty‐four mice (male, C57BL/6, 3‐month‐old) were randomly divided into two groups, namely HMP‐A and HMP‐B, with *n* = 12 in each group. OA modeling operation was performed on the right knee of each mouse. After 4 weeks of modeling, fluorescent HMPs were injected into the joint cavity to investigate the retention time of microparticles in vivo. Four time points were selected and observed on Day 1, 3, 7, and 14, respectively. Finally, the isolated knee tissue samples were collected, and fluorescence imaging technology was used to investigate the fluorescence intensity in vivo. The fluorescence intensity of HMP‐A and HMP‐B were observed at 561 nm. At the set time point, the mice were imaged by means of an IVIS at the excitation wavelength of 561 nm to determine the retention of HMPs. The radiant signals of the three groups were estimated.

### Cell Isolation and Culture

All surgical procedures were approved by the Institutional Animal Care and Use Committee of East China University of Science and Technology. Rat primary articular chondrocytes were isolated from femoral condyles and tibial plateaus of 4‐week‐old SD Rat (jsj‐lab, Shanghai, China). Articular cartilage was excised and shredded, and then digested with 0.25% trypsin‐EDTA (Sigma‐Aldrich, City of Saint Louis, USA) at 37 °C in a shaker at a speed of 200 rpm for 1 h, followed by digestion with 0.2% collagenase type II (Worthington) at 37 °C for 3 h. The supernatant was filtered through a 70 µm strainer and centrifuged to collect the cell precipitate. Cells were washed twice with PBS and cultured in DMEM/F12 supplemented with 10% fetal bovine serum (FBS; Gibco) and 1% penicillin‐streptomycin. The culture was maintained at 37 °C in a humidified atmosphere containing 5% CO2. Passages 2–5 of the chondrocytes were utilized for the in vitro experiments in this study. Primary synovium mesenchymal stem cells (SMSCs) were isolated as previously described. Briefly, synovium was collected from the joints of an 8‐week‐old male SD Rat (jsj‐lab, Shanghai, China). The synovial tissue of the rat joint was removed, cut into 2–3 mm, and added to 0.3% collagenase type V (Worthington) solution. Then, it was put in the cell incubator for 3 h and shaken it 3 times per hour. The supernatant was filtered through a 70 µm strainer and centrifuged to collect the cell precipitate. Cells were washed twice with PBS and cultured in α‐MEM supplemented with 10% fetal bovine serum (FBS; Gibco) and 1% penicillin‐streptomycin. Passages 2–5 of the SMSCs were utilized for the in vitro experiments in this study.

### Cell Biocompatibility

Live/Dead staining assay was used to evaluate the viability of cells cultured with HMPs. In brief, chondrocytes (2 × 10^4^ mL^−1^) were cultured on the lower chambers of the 24‐well plates while the HMPs were in the upper chambers. On days 1, 4, and 7, the cells were incubated with 250 µL Calcein AM/PI detection working solution (Beyotime, China) for 30 min and subsequently observed under a fluorescent microscope. The proliferation of the cells cultured with HMPs was measured using Cell Count Kit‐8 (CCK‐8, Beyotime, China). Chondrocytes were seeded in 96‐well plates at a density of 2 × 10^3^ cells well^−1^, attached overnight in DMEM/F12 containing 10% FBS, 100 U mL^−1^ penicillin, and 100 µg mL^−1^ streptomycin. Three groups of Ctrl, HMP‐A, and HMP‐B were divided in a 96‐well plate. All samples were tested in sextuplicate, and the results were normalized to control and expressed as mean ± SD.

### Recruitment and Migration Assay of SMSCs In Vitro

HMPs and SMSCs, fibroblasts, and macrophages were co‐cultured in matrix glue respectively using a 96‐well plate with a black glass bottom. Hundred microliters of matrix gel, 2 × 10^3^ cells, and Rhodamine labeled HMPs were added to each well. The cells were fixed after 24 h of culture, and immunofluorescence staining was performed. Finally, the recruitment of SMSCs was observed by a laser confocal microscope.

SMSC migration was assessed using transwell inserts containing a polycarbonate membrane (Corning, New York, NY, USA). The HMPs were placed in the bottom chamber of the wells, and SMSCs were seeded on top of the inserts. The culture medium was added to the top insert and bottom chamber. After a 24‐hour incubation at 37 °C, the cells remaining on the top of the inserts were removed, while those that had migrated to the lower side of the insert were fixed with cold methanol and stained with crystal violet (0.1% w/v). The migrated cells were then imaged by a light microscope and quantified.

### Chondrogenesis of SMSCs In Vitro

Chondrogenic differentiation was induced in a commercial chondrogenic differentiation medium of synovial mesenchymal stem cells (GUXMX‐90041, OriCell), with the supplementation of transforming growth factor‐β3 (TGF‐β3). Pellets were formed by centrifuging 1 × 10^5^ of SMSCs at 300 g for 5 min in 500 µL of differentiation medium in 15 mL centrifuge tubes, followed by 24‐hour incubation at 37 °C. Then the pellets were co‐cultured with HMP‐A, HMP‐B, and dmHMPs for 14 days at 37 °C in a humidified incubator with 5% CO2, with media changes every 2 days. After 14 days, the pellets were sliced into 4‐µm‐thick sections and subjected to toluidine blue, Alcian blue, and immunofluorescence staining.

### Cell Immunofluorescence

In vitro cell experiments, the expression of Col II and Sox‐9 of synovial stem cells was investigated by co‐culture of microparticles and synovial stem cells. Then, the analysis was performed by immunofluorescence. In brief, the cells were fixed with 4% paraformaldehyde for 15 min, then permeated with 0.1% Triton X‐100 for 15 min, and blocked with 5% goat serum at room temperature for 1 h. The cells were incubated overnight at 4 °C with the antibodies Col II and Sox‐9. Primary antibodies were then visualized with species‐appropriate secondary antibodies. The cytoskeleton was stained by FITC – phalloidin (Thermo Fisher) for 45 min and the nuclei were stained with DAPI (Thermo Fisher) for 15 min. A Leica confocal microscope (Leica, TCS SP8) was used to image samples. Immunofluorescence was also used to investigate CD197 and CD206 in the co‐culture of macrophages and microparticles. The antibodies from the above steps were changed to CD197 and CD206 incubated overnight, and the rest of the steps were the same.

### Flow Cytometry

For flow cytometric analysis of macrophages, the knee joints of the mouse were removed from euthanized mice after thoroughly removing the adherent muscles and then crushed in ice‐cold PBS with a mortar and pestle. To obtain all the cell suspension inside the knee joint, samples were digested at 37 °C for 15 min with 3 mg mL^−1^ collagenase type I (Worthington), 2 mg mL^−1^ collagenase type II (Worthington), 4 mg mL^−1^ dispase (Roche Diagnostics), and 1 U mL^−1^ DNAse I (Sigma) in HBSS with calcium and magnesium. The digested cells were then transferred into HBSS with 2 mm EDTA to stop the enzymatic reaction. After centrifugation, the cells were blocked with antiCD16/32 antibody for 20 min at 4 °C, followed by staining with 7‐AAD‐APC‐Cy7, anti‐CD45‐BV510, anti‐F4/80‐APC, anti‐CD11b‐BV421, anti‐CD197‐PE, for 45 min at 4 °C. Before staining with antiCD206‐AF700, the cells were permeabilized using a Cyto‐FastTM Fix/Perm Buffer Set (Biolegend). After washing with HBSS for several times, the cell suspension was then assessed on a CytoFLEX flow cytometer system (Beckman) and analyzed using FlowJo software (Tree Star).

### Western Blots Analysis

Total protein extracts were obtained via the lysis of Macrophages treated in cold radioimmunoprecipitation assay (RIPA) buffer containing phenylmethylsulfonyl fluoride. Cell lysates were equilibrated to equal concentrations with loading buffer and boiled for 10 min. Subsequently, equal concentrations of the samples were separated in a 4–15% SDS‐PAGE precast gel (Beyotime) and then blotted on polyvinylidene fluoride membranes (Millipore). The membranes were incubated with specific antibodies for M2 macrophages polarization protein STAT3, phosphorylated STAT3(p‐STAT3), STAT6, and phosphorylated STAT6 (p‐STAT6) antibodies followed by horseradish peroxidase HRP‐conjugated secondary antibodies. β‐actin was used as a loading control. The protein bands were visualized using a chemiluminescence imaging system (Tanon, Shanghai, China).

### Mouse OA Model

All procedures followed the National Institute of Health Guide for the Care and Use of Laboratory Animals and were approved by the East China University of Science and Technology (approved number: ECUST‐21041). For the experimental OA model, a surgical procedure was performed in 3‐month‐old mice (young) and 18‐month‐old mice (aged). Under general anesthesia with isoflurane, Destabilization of the Medial Meniscus (DMM) was surgically performed by transection of the medial meniscus tibial ligament in the right knee joints using a surgical microscope. Sham operations were also performed by opening and exposing the structures of the right knee and then closing the skin incision without disturbing the joint tissue. These mice were randomly divided into four groups (Sham, Ctrl, HMP‐A, and dmHMPs) after 4 weeks, and treated as follows: Sham (No treated), Ctrl (10 µL of PBS intra‐articular injected), HMP‐A (10 µL of HMP‐A intra‐articular injected), dmHMPs (10 µL of HMP‐A and HMP‐B intra‐articular injected). Intra‐articular injections were given every 2 weeks, and knee joint samples were collected at the 8th week. (*n* = 5 for each group)

### Micro‐CT Analysis

Micro‐CT analysis was conducted on a Scano Medical µCT 35 system using the previously described parameters.^[^ ^60^ ^]^ The resolution of 18 µm was used to scan the femur and got the 8‐bit image of all samples. Then the AVIZO software was used to reconstruct each knee joint.

### Histology, Immunofluorescence and Immunohistochemistry

For histological analysis, the knee joint samples were fixed with 4% neutral paraformaldehyde, decalcified with 0.5 m EDTA, dehydrated with ethanol, and then embedded in paraffin. The samples were then sectioned into 4.5 µm pieces. Subsequently, H&E staining, Toluidine blue staining, and Safranin O‐Fast Green staining were conducted to investigate the cartilage and synovium, respectively. For immunofluorescence analysis, knee joint samples were retrieved from the mice and snap‐frozen in an optimal cutting temperature medium. Immunofluorescence staining and analysis were performed as described previously. Knee joint sections were cut using a cryotome, mounted on slides, and stained with different primary antibodies: LepR, Sox‐9, CD197, CD206, F4/80, Acan, Col X, Col II, and Ihh overnight at 4 °C. Primary antibodies were then visualized with species‐appropriate secondary antibodies. The sections were mounted by ProLong Gold Antifade reagent with DAPI (Cell Signaling Technology). The sections were analyzed with a laser confocal microscope (Leica, TCS SP8). For immunohistochemistry analysis, the sections were incubated with primary antibodies against Col I, Col II, and MMP‐13 overnight at 4 °C, followed by a 1‐hour incubation with the corresponding secondary antibodies. Afterward, the paraffin sections were stained with 3,3′‐diaminobenzidine (DAB) substrate. The sections were analyzed with an inverted optical microscope (Leica, DMI‐8). ImageJ software was used to quantify the relative expressions of Col I, Col II, and MMP‐13.

### Gait Acquisition

The fore paws of the mice were stained with red ink, and the hind paws were stained with blue ink. Subsequently, the mice were guided to walk naturally on the paper to collect footprints. At least four to six consecutive and clear footprints from each mouse were used for data analysis. Typically, the initial few footprints were excluded from statistical analysis as the animals might not have achieved a steady gait at this stage.

### Statistical Analysis

Results were analyzed using GraphPad Prism software (version 9.2; GraphPad, La Jolla, CA, USA). All data were expressed as means ± SD. N numbers indicated biological replicates of experiments performed at least three times unless otherwise indicated. Unpaired, two‐tailed Student's *t*‐tests were used for comparisons between the two groups. For comparison of multiple experimental groups, either one‐way analysis of variance (ANOVA) or two‐way ANOVA was performed where indicated. A *P*‐value of 0.05 was considered statistically significant: *P < 0.05, **P < 0.01, ***P < 0.005, and ****P < 0.001.

## Conflict of Interest

The authors declare no conflict of interest.

## Supporting information



Supporting Information

## Data Availability

The data that support the findings of this study are available in the supplementary material of this article.
