# Supplementary material for: Dual‐Modular Hydrogel Microparticles with Precision‐Modulation of Inflammatory Microenvironment Dictate Full‐Thickness Cartilage Regeneration for Osteoarthritis Repair
Source: Adv Sci (Weinh). 2025 Jun 29;12(36):e04965. doi: 10.1002/advs.202504965 (PMC12462947; doi:10.1002/advs.202504965)
Supplement: Supplementary file 1 — Supporting Information [file ADVS-12-e04965-s001.docx]

Supporting Information

Dual-modular hydrogel microparticles with precision-modulation of inflammatory microenvironment dictate full-thickness cartilage regeneration for osteoarthritis repair

*Xinye Chen, Yuanman Yu*, Zirui He, Lina Pan, Jing Wang*, and Changsheng Liu**


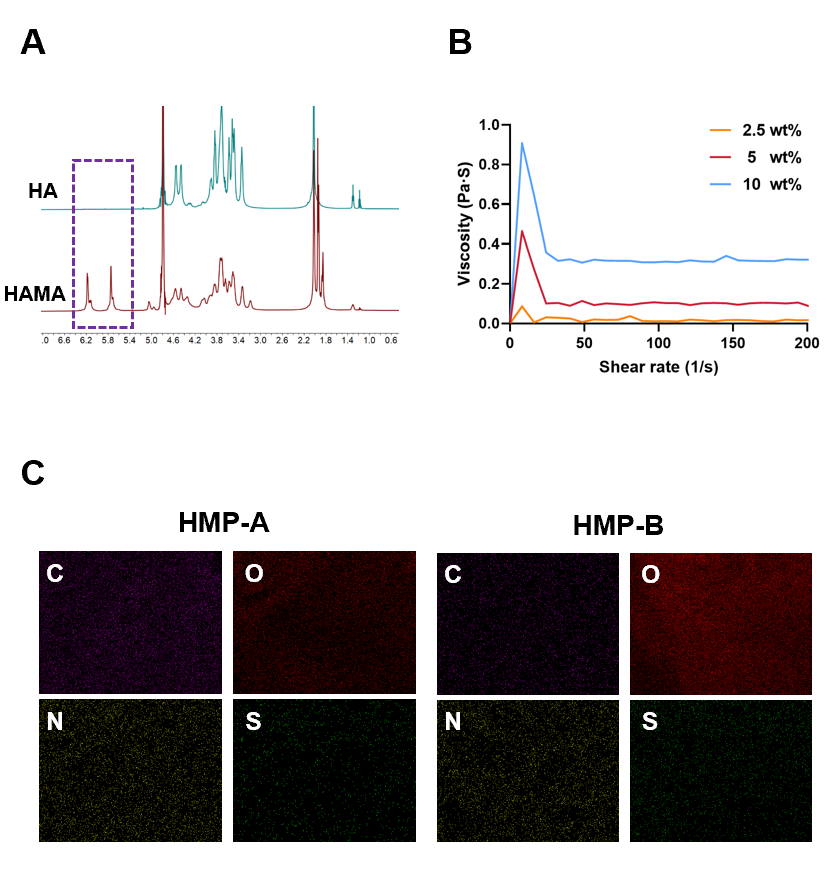


**Figure S1. Physical properties of synthetic HAMA and HMP-A and HMP-B.** A) ^1^H NMR analysis of HAMA and HA. B) Rheological curves of HAMA solutions with different concentrations (2.5 wt%, 5 wt% and 10 wt%). C) Elemental mapping images of the HMP-A and HMP-B. C: Carbon, O: Oxygen, N: Nitrogen, S: Sulfur.


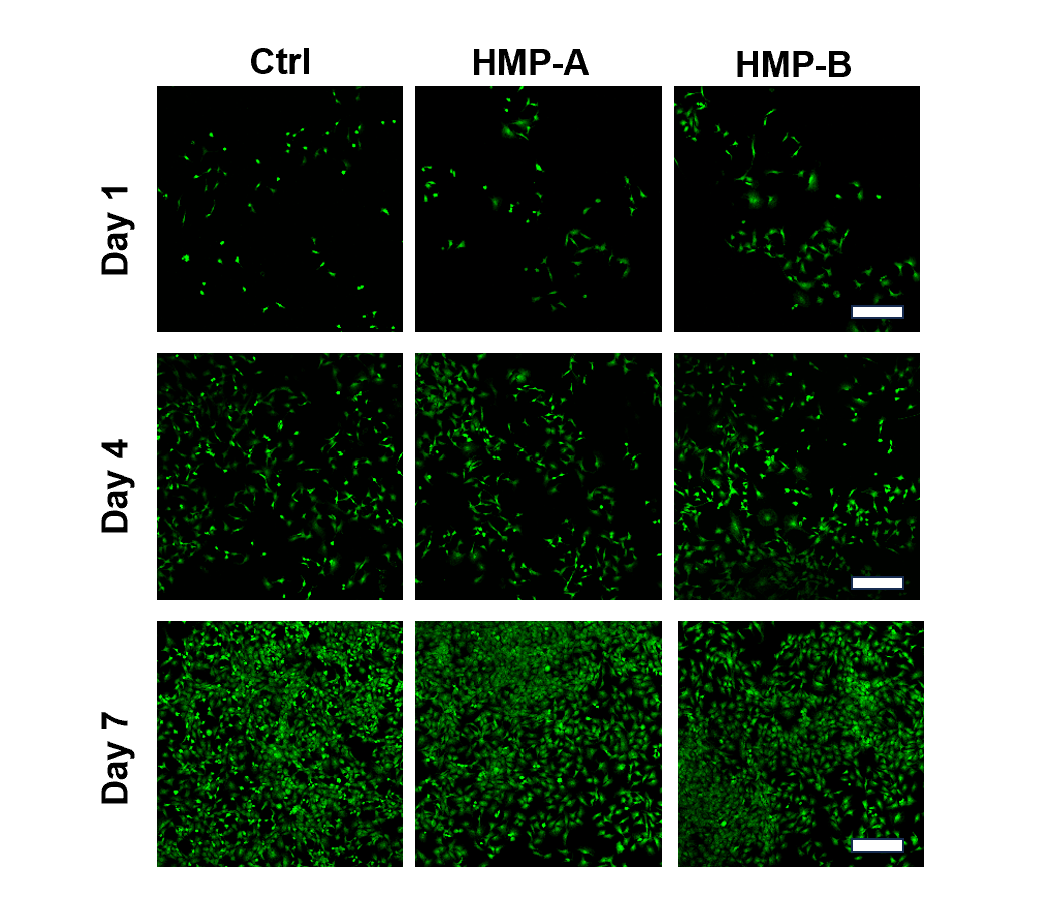


**Figure S2. In vitro biocompatibility of HMP-A and HMP-B.** Live (green)/Dead (red) fluorescence results of Ctrl, HMP-A, and HMP-B groups on 1, 4, and 7 days. Scale bar: 200 μm.


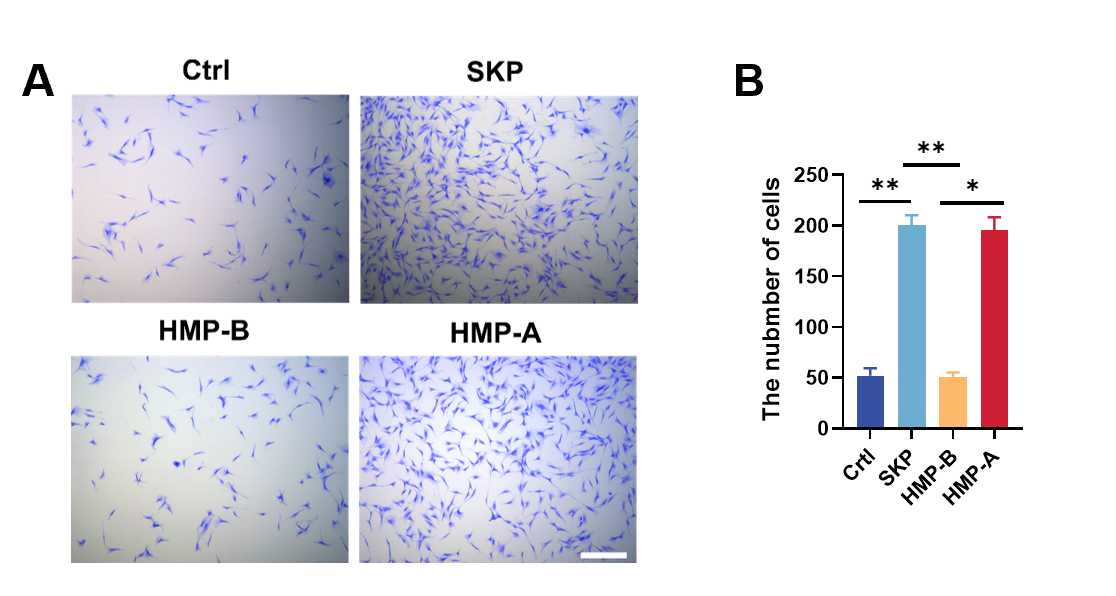


**Figure S3.** **Transwell assay to monitor SMSC recruitment in vitro.** A) Representative images of Transwell bottom membrane stained with crystal violet after 24-hour culture. Scale bar: 200 μm. B) Quantification of cells observed at the bottom of the membrane (n = 3). Data are shown as means ± SD. Statistical analysis was performed using one-way ANOVA with Tukey’s post hoc test. *P < 0.05, **P < 0.01, ***P < 0.005, and ****P < 0.001; ns, not significant.


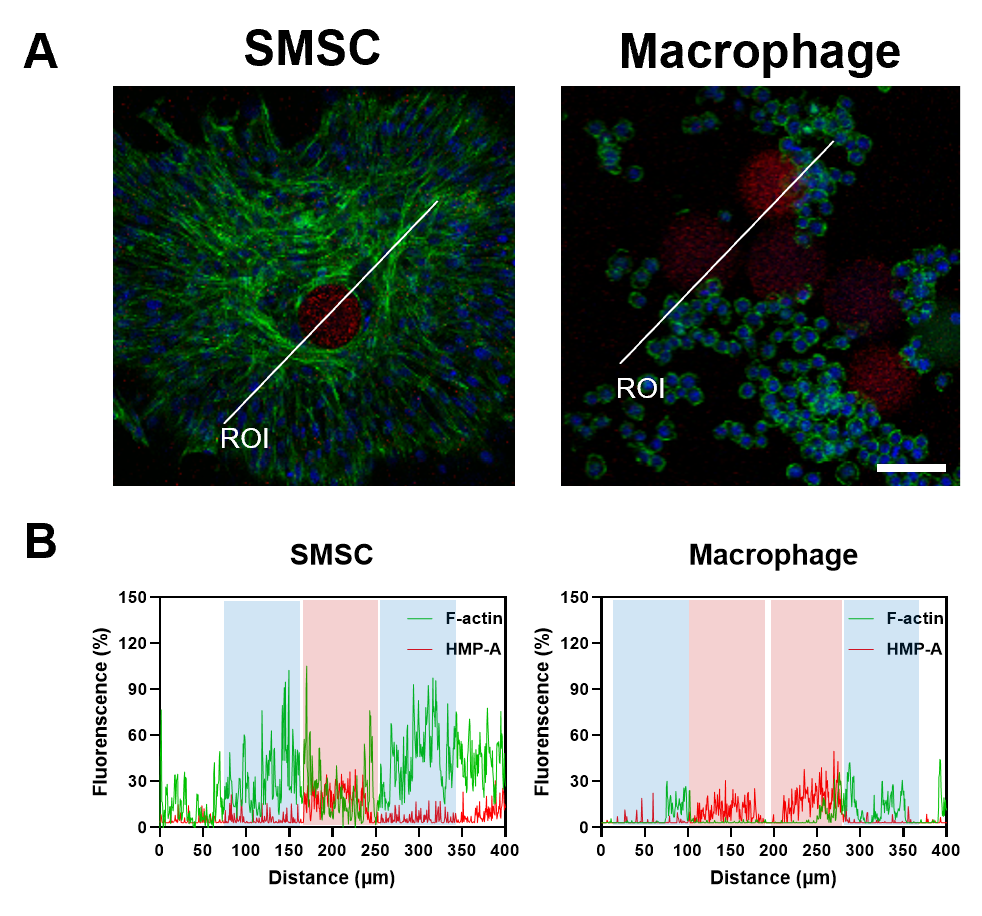


**Figure S4. Analysis of HMP-A's Selective Recruitment of SMSC.** A) Representative immunofluorescence images of HMP-A co-cultured with SMSCs and macrophages. Scale bars, 100 μm. B) Quantitative analysis of the selective recruitment of SMSCs by HMP-A. The regions delineated by the red rectangle correspond to the fluorescence emission of HMP-A. The regions delineated by the blue rectangle correspond to the fluorescence emission of SMSC and Macrophage.


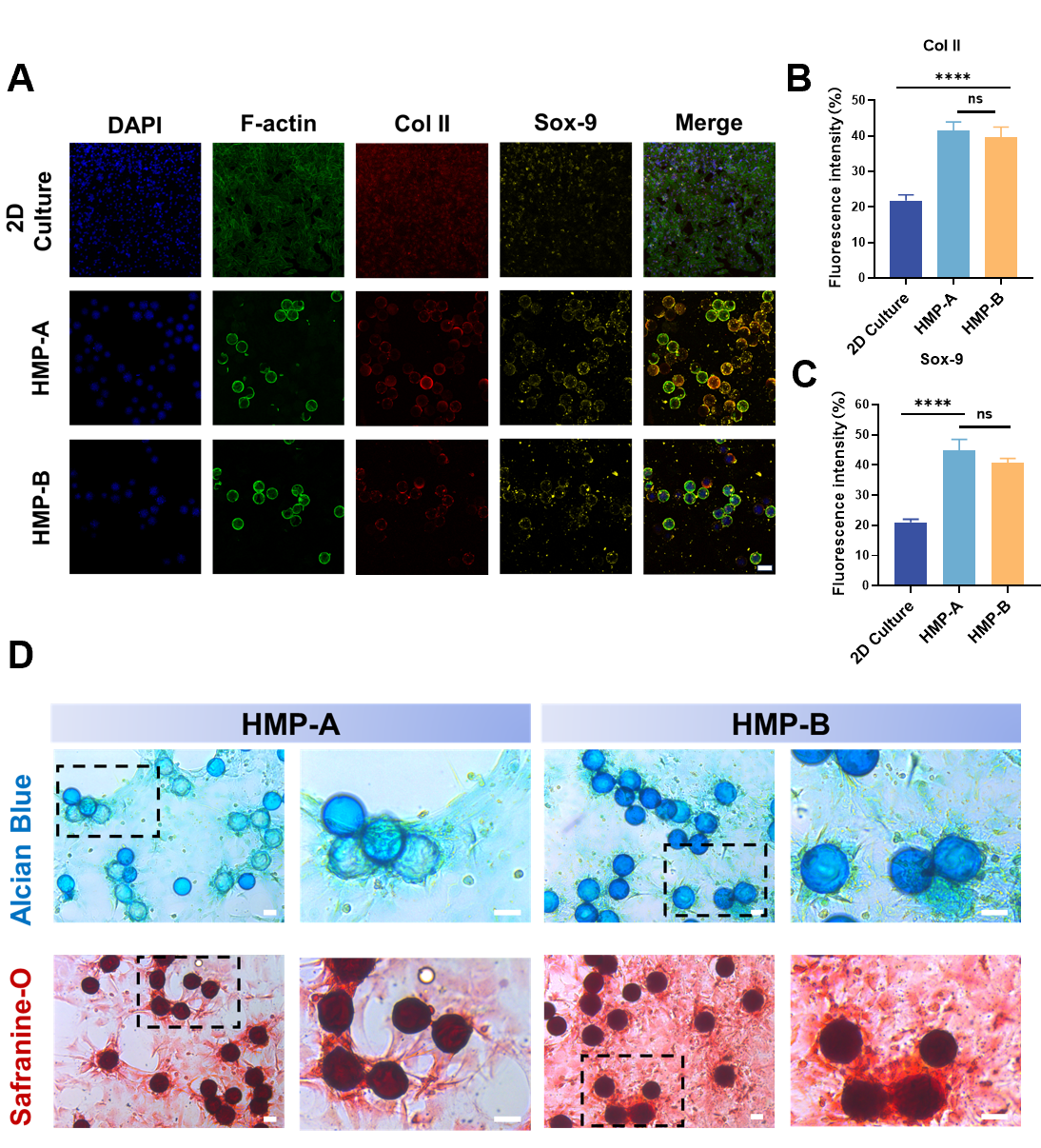


**Figure S5.** The three-dimensional structure of HMP-A and HMP-B promotes chondrogenic differentiation of SMSCs. A) Representative images of Col II and Sox-9 immunostainings of SMSCs cultured on 2D Culture, HMP-A and HMP-B for 7 days. Scale bar: 100 μm. B, C) Quantification of fluorescence intensity of Col II and Sox-9 (n = 3). D) Alcian blue staining and Safranine-O staining of SMSCs cultured on HMP-A and HMP-B for 7 days. Scale bar: 100 μm. Data are shown as means ± SD. Statistical analysis was performed using one-way ANOVA with Tukey’s post hoc test. *P < 0.05, **P < 0.01, ***P < 0.005, and ****P < 0.001; ns, not significant.


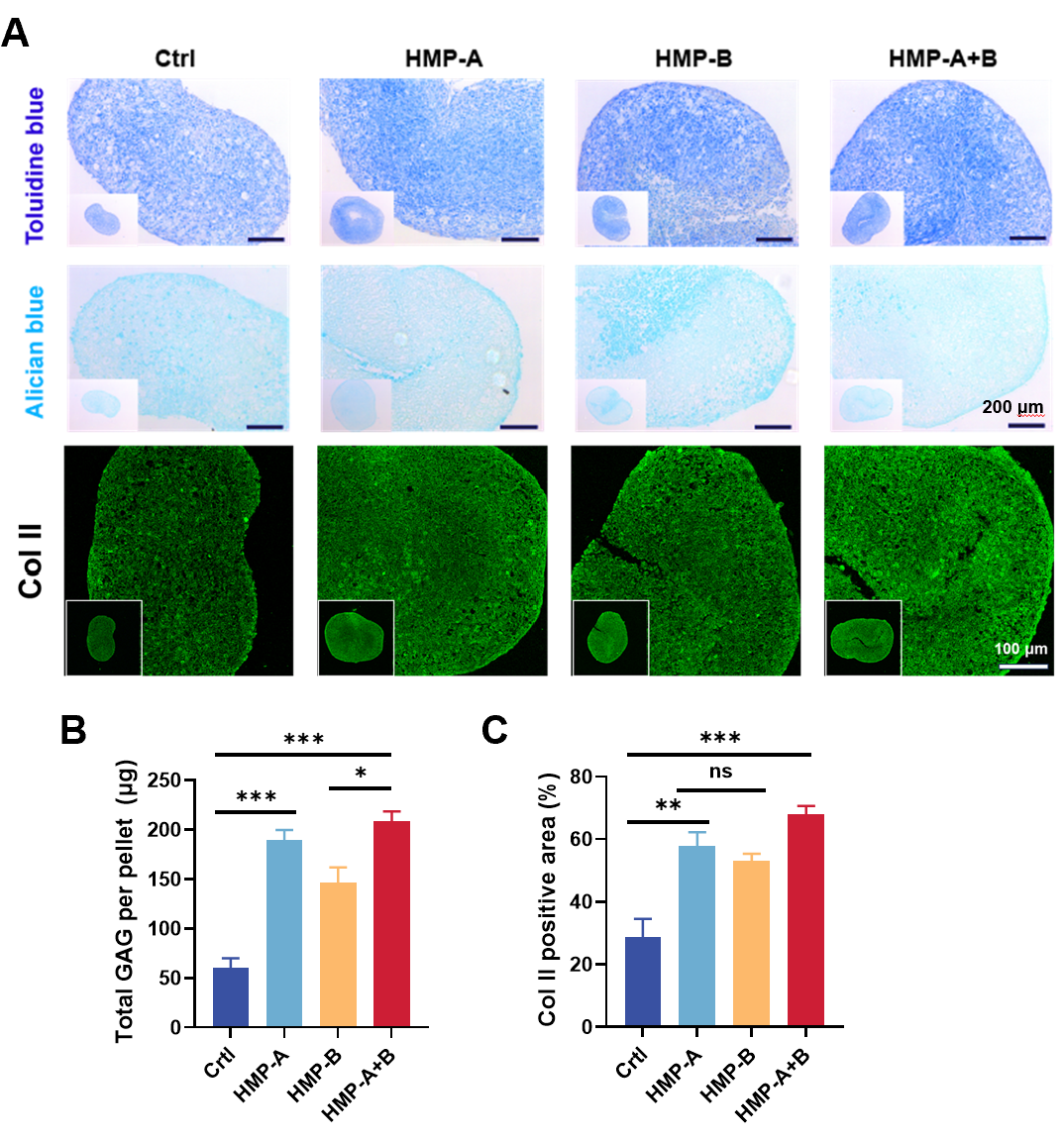


**Figure S6. SMSCs and HMPs were co-cultured to form SMSCs cartilage pellets after 14 days.** A) Toluidine blue staining and Alcian blue staining of pellets. Scale bars, 200 μm. Immunofluorescence staining of Col II in pellets. Scale bars, 100 μm. B) Quantitative analysis of total GAG content per pellet (n = 3). C) Quantitative analysis of Col II positive area in pellets (n = 3). Data are shown as means ± SD. Statistical analysis was performed using one-way ANOVA with Tukey’s post hoc test. *P < 0.05, **P < 0.01, ***P < 0.005, and ****P < 0.001; ns, not significant.


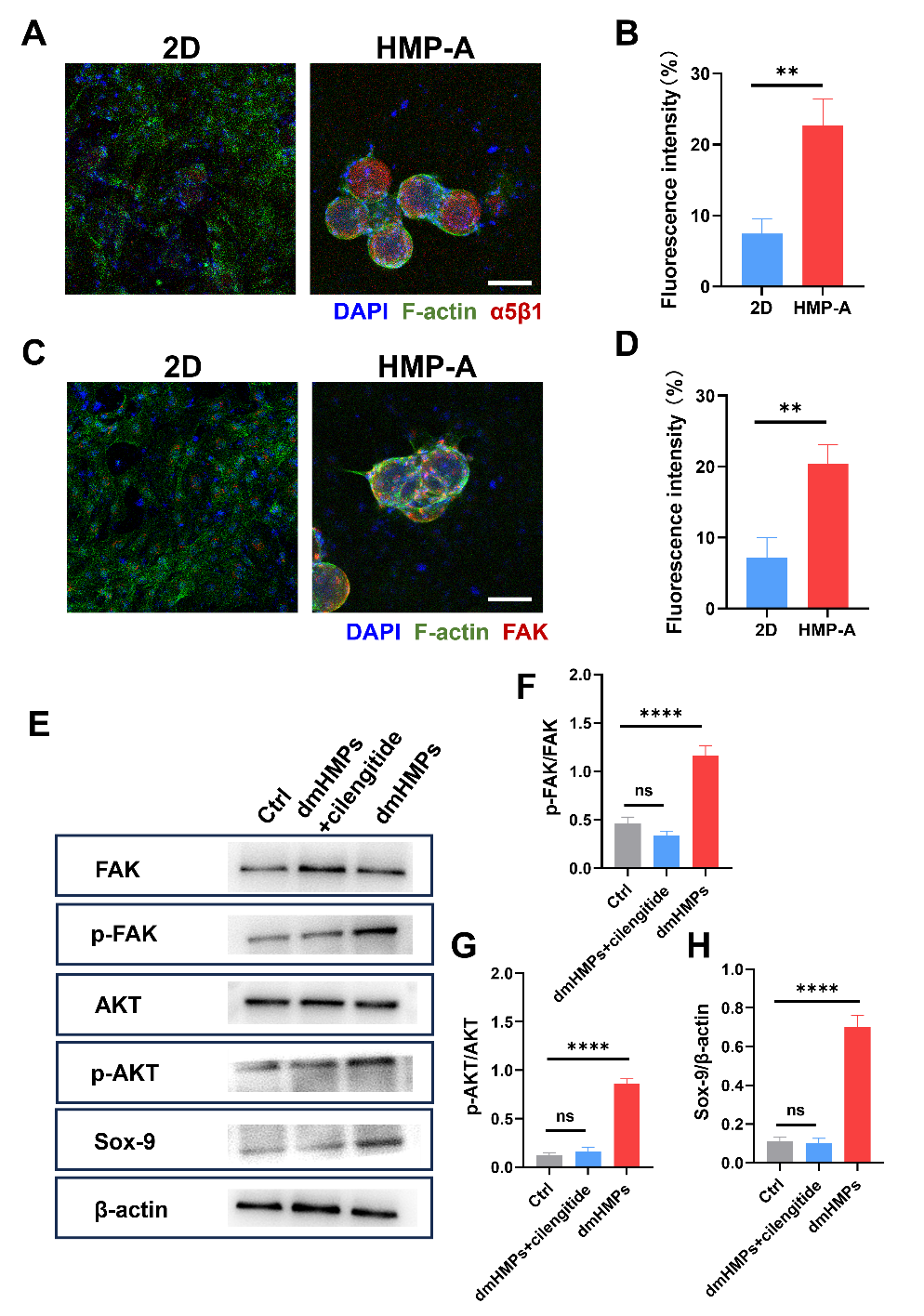


**Figure S7. dmHMPs regulate the biological functions of SMSCs through integrin α5β1.** A) Immunofluorescence staining of α5β1 in SMSCs. Scale bars, 100 μm. B) Quantitative analysis of the fluorescence intensity of α5β1 (n = 3). C) Immunofluorescence staining of FAK in SMSCs. Scale bars, 100 μm. D) Quantitative analysis of the fluorescence intensity of FAK (n = 3). E) Representative images of Western blots of the phosphorylation of FAK and AKT of SMSCs treated with dmHMPs for 24 hours. Cilengitide: an integrin α5β1 inhibitor. F-H) Quantitative analysis of p-FAK/FAK, p-AKT/AKT and Sox-9/β-actin (n = 3). Data are shown as means ± SD. Statistical analysis was performed using unpaired two-tailed Student’s *t* test and one-way ANOVA with Tukey’s post hoc test. *P < 0.05, **P < 0.01, ***P < 0.005, and ****P < 0.001; ns, not significant.


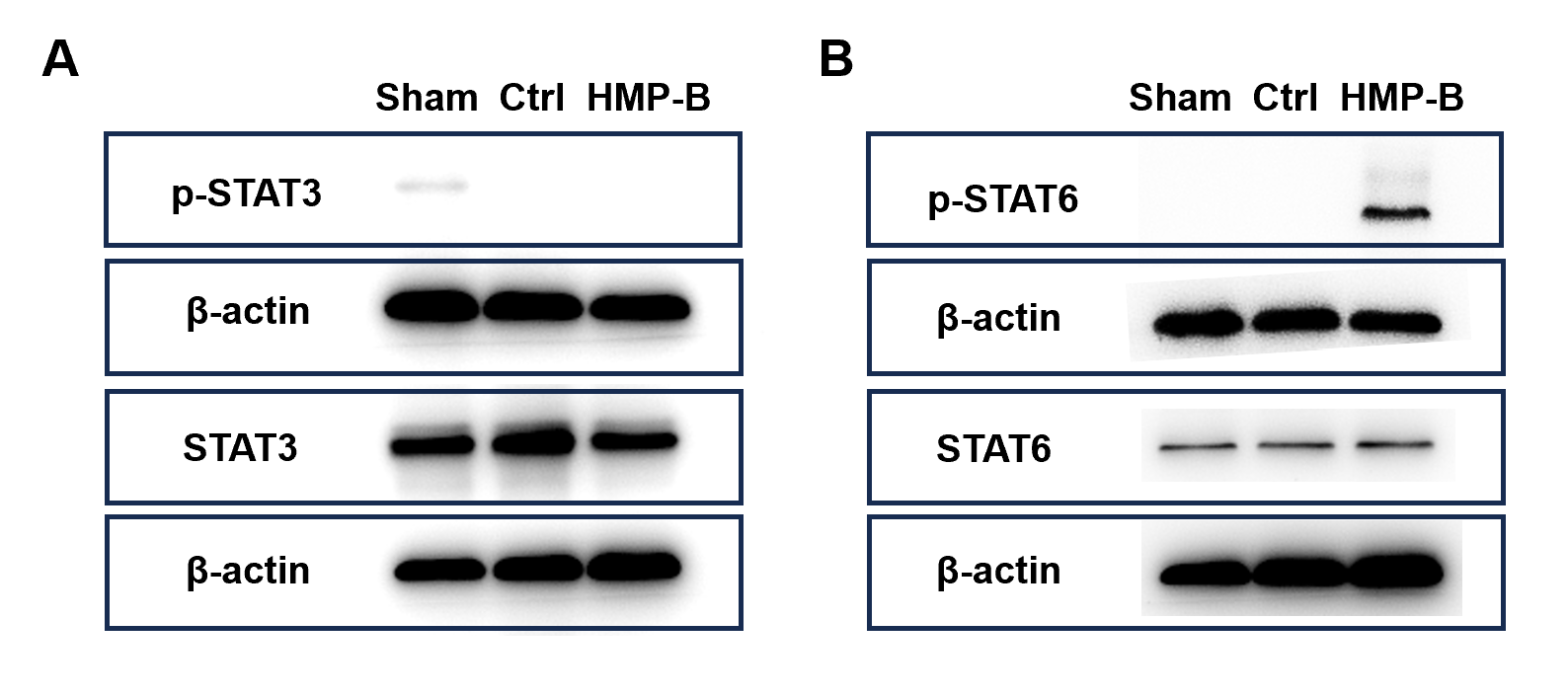


**Figure S8.** **Analysis of HMP-B promoting the polarization of macrophages from M1 phenotype to M2 phenotype.** A) Representative images of Western blots of the phosphorylation of Stat3 of macrophages treated with HMP-B for 24 hours. B) Western blots of the phosphorylation of Stat6 of macrophages treated with HMP-B for 24 hours. (n = 3).

**Table S1. Osteoarthritis Research Society International (OARSI) scores**

| Grade | Osteoarthritic damage |
| --- | --- |
| 0 | Normal |
| 0.5 | Loss of Safranin-O without structural changes |
| 1 | Small fibrillations without loss of cartilage |
| 2 | Vertical clefts down to the layer immediately below the superficial layer and some loss of surface lamina |
| 3 | Vertical clefts/erosion to the calcified cartilage extending to <25% of the articular surface |
| 4 | Vertical clefts/erosion to the calcified cartilage extending to 25-50% of the articular surface |
| 5 | Vertical clefts/erosion to the calcified cartilage extending to 50-75% of the articular surface |
| 6 | Vertical clefts/erosion to the calcified cartilage extending >75% of the articular surface |

**Table S2. Synovitis score**

| Grade | Enlargement of synovial lining cell layer |
| --- | --- |
| 0 | The lining cells form one layer |
| 1 | The lining cells form 2–3 layers |
| 2 | The lining cells form 4–5 layers, few multinucleated cells might occur |
| 3 | The lining cells form more than5 layers, the lining might be ulcerated and multinucleated cells might occur |
| Grade | Density of the resident cells |
| 0 | The synovial stroma shows normal cellularity |
| 1 | The cellularity is slightly increased |
| 2 | The cellularity is moderately increased, multinucleated cells might occur |
| 3 | The cellularity is greatly increased, multinucleated giant cells, pannus formation and rheumatoid granulomas might occur |
| Grade | Inﬂammatory inﬁltrate |
| 0 | No inﬂammatory inﬁltrate |
| 1 | Few mostly perivascular situated lymphocytes or plasma cells |
| 2 | Numerous lymphocytes or plasma cells, sometimes forming follicle-like aggregates |
| 3 | Dense band-like inﬂammatory inﬁltrate or numerous large follicle-like aggregates |
| Sum 0 or1 | No syvitis |
| Sum 2 - 4 | Low-grade synovitis |
| Sum 5 - 9 | High-grade synovitis |
